# Work addiction and social functioning: A systematic review and five meta-analyses

**DOI:** 10.1371/journal.pone.0303563

**Published:** 2024-06-04

**Authors:** Viktória Kenyhercz, Barbara Mervó, Noémi Lehel, Zsolt Demetrovics, Bernadette Kun

**Affiliations:** 1 Institute of Psychology, ELTE Eötvös Loránd University, Budapest, Hungary; 2 Doctoral School of Psychology, ELTE Eötvös Loránd University, Budapest, Hungary; 3 Centre of Excellence in Responsible Gaming, University of Gibraltar, Gibraltar, Gibraltar; 4 College of Education, Psychology and Social Work, Flinders University, Adelaide, Australia; Universita Cattolica del Sacro Cuore Sede di Roma, ITALY

## Abstract

As theoretical models suggest, work addiction has several adverse correlates and consequences, such as unfavorable personality traits, physical and psychological symptoms, and social conflicts. Both early and recent concepts emphasize that individuals with work addiction have more problematic social life due to obsessive overwork. This includes negative impacts on family, workplace, and other relationships. The present study aimed to systematically review and meta-analyze all the empirical studies that examined the association between work addiction and any dimension of social life, as such an analysis has never been conducted before. Studies published from 1995 to 2022 were identified through a systematic search. 102 eligible studies were included in the review, with 75 studies contributing to five different meta-analyses. The results indicated significant associations between work addiction and: (1) lower work-life balance, (2) reduced social functioning, and increased difficulties in (3) family relationships, (4) intimate relationships, and (5) relationships with the community, friends, and colleagues. The associations were found to be independent of gender and age. The meta-analytic study highlights research gaps in the field and suggests future directions, including exploring attachment styles and early social relationships in work addiction, investigating the association between social and emotional competencies and work addiction, examining the role of escape motivation, and exploring the characteristics of the partners (spouses) of workaholics. Since the quality of social relationships and social support are crucial factors in physical and mental health, the prevention and intervention of work addiction should be prioritized in organizational and clinical settings.

## Introduction

### Work addiction

Based on three representative population studies conducted in Norway, Hungary, and Germany, 8–10% of the employed population is affected by work addiction in the western culture [1–3]. Moreover, the prevalence of work addiction in South Korea is much higher, namely 39.7% [4]. In alignment with this high populational involvement, the topic of work addiction is in the limelight of research [5]. Initially, Oates [6] was using the term ‘workaholism’ in the early 1970s. He proposed that workaholism is driven by an uncontrollable need to work constantly, so the affected individual is continuously thinking about work, even when working hours are over, and the person works more than the employer usually expects [6]. Since then, the term ‘workaholic’ has become a frequently used phrase describing individuals who work ‘too much’. On the other hand, researchers generally use both terms [workaholism and work addiction] interchangeably, and as such it shows the confusion and lack of consensus of definition. Griffiths and colleagues [5] suggested differentiating between workaholism and work addiction, namely, ‘workaholism’ is a term that appears in the literature in a more positive way [5], and it was also defined as the *„best-dressed mental health problem of the twentieth century”* [7, p. 2]. Hence “work addiction” is a more concrete psychological construct and it fits more into the addiction framework [5]. Research on behavioral addictions has made significant progress in recent decades, contributing to the conceptual validity of certain disorders such as gambling disorder and gaming disorder [8]. Work addiction is often referred to as a behavioral addiction, however, it is still not included in the DSM-5 [9], or the ICD-11 [10].

According to the existing literature, research on work addiction has been examined from different approaches, such as by measuring *time spent with work* [11] or *inner drives and needs* [12]. Ng and colleagues [13] defined work addiction as a *behavioral pattern* that includes excessive amount of time and effort investment in work in combination with obsession with work. According to them, individuals who are at risk of work addiction tend to devote extensive hours to work, often sacrificing their personal time as a result of their compelling obsession. A new conceptualization of work addiction that encompasses cognitive, emotional, motivational, and behavioral dimensions of the problem was proposed by Clark and colleagues [14]. These authors described work addiction with the following four elements 1) compulsive motivation to work (i.e., motivational factor), 2) thinking of work persistently and in an uncontrollable way (i.e., cognitive factor), 3) the presence of negative emotions if working is not possible (i.e., emotional factor), and 4) excessive work that is more than expected or required (i.e., behavioral factor) [14]. The strength of this new concept is emphasizing the main psychological processes in the background of work addiction, however, this dimensional model does not include all the symptoms regarding the addictive nature of the problem. According to Griffiths and Karanika-Murray [15], work addiction shows the same components as other substance-related and other addictions, namely: 1) salience, 2) tolerance, 3) mood modification, 4) relapse, 5) withdrawal, 6) intrapersonal and interpersonal conflicts, and 7) problems caused by the compulsive behavior.

### Work addiction and social relationships in theoretical models

The negative impact of work addiction on one’s social relationships has been highlighted for a long time. It was already stated in Oates’ early definition that workaholics are those people for whom “*need for work has become so excessive that it creates noticeable disturbance or interference with his bodily health*, *personal happiness*, *and interpersonal relations*, *and with his smooth social functioning”* [6, p.4.]. This definition is in line with the components model of addictions [16, 17] that emphasizes impaired social functioning regarding two components: 1) "intrapersonal conflicts” refer to difficulties in balancing the individual’s professional and personal life ending in regular conflicts with family members, relatives, friends, and important others; and 2) “problems” reflects the neglect of social life or losses of important relationships because of excessive and compulsive work. These theoretical concepts are in accordance with the clinical therapist Robinson’s observations who was one of the first authors to develop a measure for assessing the risk of work addiction [18]. Based on his family therapist experiences, Robinson [19] listed the “*difficulty with relationships”* as an indicator of work addiction in his conceptualization. As Robinson [7] argued, as thoughts and energies of a person suffering from work addiction are always related to work, people surrounding them tend to feel neglected due to the lack of attention they receive. As a result, individuals with work addiction have poorer family relationships, and family members of workaholics more often show symptoms of mental problems [20]. Years later, Schaufeli and colleagues [21] developed a model of work addiction that highlighted two primary components: excessive work and compulsive work. According to the model, both excessive and compulsive work exert a significant and negative influence on personal life and social relationships. Workaholics tend to spend significantly more time at work than with friends, engaging in entertainment, or attending social events. They find it particularly challenging to allocate their free time away from work.

In another comprehensive model of work addiction, Loscalzo and Giannini [22] proposed that work addiction is a clinical condition containing internalizing and externalizing symptoms, and these authors suggested using DSM-like criteria for work addiction, as with other addictive disorders. Among the twelve criteria, three contain problems in social functioning, namely: 1) „recurrent work-related behaviors as described above resulting in a failure to fulfill major role obligations at home”; 2) „continued work-related behaviors as described above despite having persistent or recurrent social or interpersonal problems caused or exacerbated by these behaviors themselves”; and 3) „important social, family, or recreational activities are given up, reduced or impaired because of workaholism” [22, p. 321.]. Taking a closer look at the empirical models that emphasize strong connection between work addiction and difficulties in social functioning, we should also mention the transactional model [23]. This model argues that coping as one’s choice is determined by primary appraisals and secondary appraisals. McMillan and colleagues [24] formulated the coping model of work addiction, and they highlighted that the partner of the individual with work addiction could act as a stress-buffer, helping the person to cope with stress factors. In addition, they suggested that work addiction can be characterized by denial and relationship distress as well [24], thus work addiction has damaging side effects on one’s health and social relationships. Research on family systems aims to develop models on the relationship between family functioning and dysfunctional behavior (i.e., work addiction), and these models suggest that the problem is located rather in the family system than in the person [24].

During the last decades, several research investigated the correlates, risk factors, and possible consequences of work addiction. At the same time, most of the theoretical concepts of work addiction emphasize the harmful effect of work addiction on the quality of social life. Although one meta-analytic study has already investigated the relevant correlates of work addiction including some of the social factors [25], a comprehensive review that incorporates all the social life characteristics of work addiction is still missing. The meta-analysis conducted by Clark et al. [25] focused only on a narrower aspect of the research question. Their aim was to comprehensively analyze the correlates of work addiction, which led them to include only certain aspects of social relationships (i.e., family satisfaction/functioning, marital disaffection, and work-life conflict). It is important to note that their search concluded eleven years ago, in 2013, and thus many recent articles have not been included in their analysis. Additionally, the authors did not provide specific details about each individual article included, making it unclear how many relevant studies on social relationships were used for their analysis. However, it is known that the meta-analysis included three articles to analyze marital disaffection, six articles to analyze relationship satisfaction, and 22 articles to analyze work-life conflict. Our review, on the other hand, aimed to incorporate all empirical work relevant to social relationships and analyze it in a narrative manner. By adopting this approach, we can obtain a more up-to-date and comprehensive understanding of the social relationship experiences of individuals affected by work addiction.

### Aim of the current study

One of the aims of the present paper was to systematically review the literature that examined *any dimensions of social life* in connection with work addiction. This is planned to be explored both in the broader sense, which includes issues of work-life balance and social functioning, but also at a more concrete level, which refers to specific social relations. In the latter, we plan to examine all social relationships, including family life and couple relationships in the private sphere, as well as broader community, friendship, and workplace relationships. Thus, our aim is to examine six broad themes: (1) work-life balance, (2) general social functioning, (3) actual family life, (4) family of origin and offspring of workaholics, (5) intimate relationship, and (6) other relationships i.e., relationships with friends, community, and colleagues. Our further aim was to conduct meta-analyses of the results published so far. Based on the theoretical models of work addiction [7, 13, 14, 17, 21, 22], we expected that individuals affected by work addiction have more conflicts between work and life, work and family, and have poorer social relationships. In sum, the main goal of the current systematic review is to compare research findings with empirical models, and to verify or reject the assumption on the relationship between work addiction and problematic social life.

## Materials and methods

**Fig 1:**
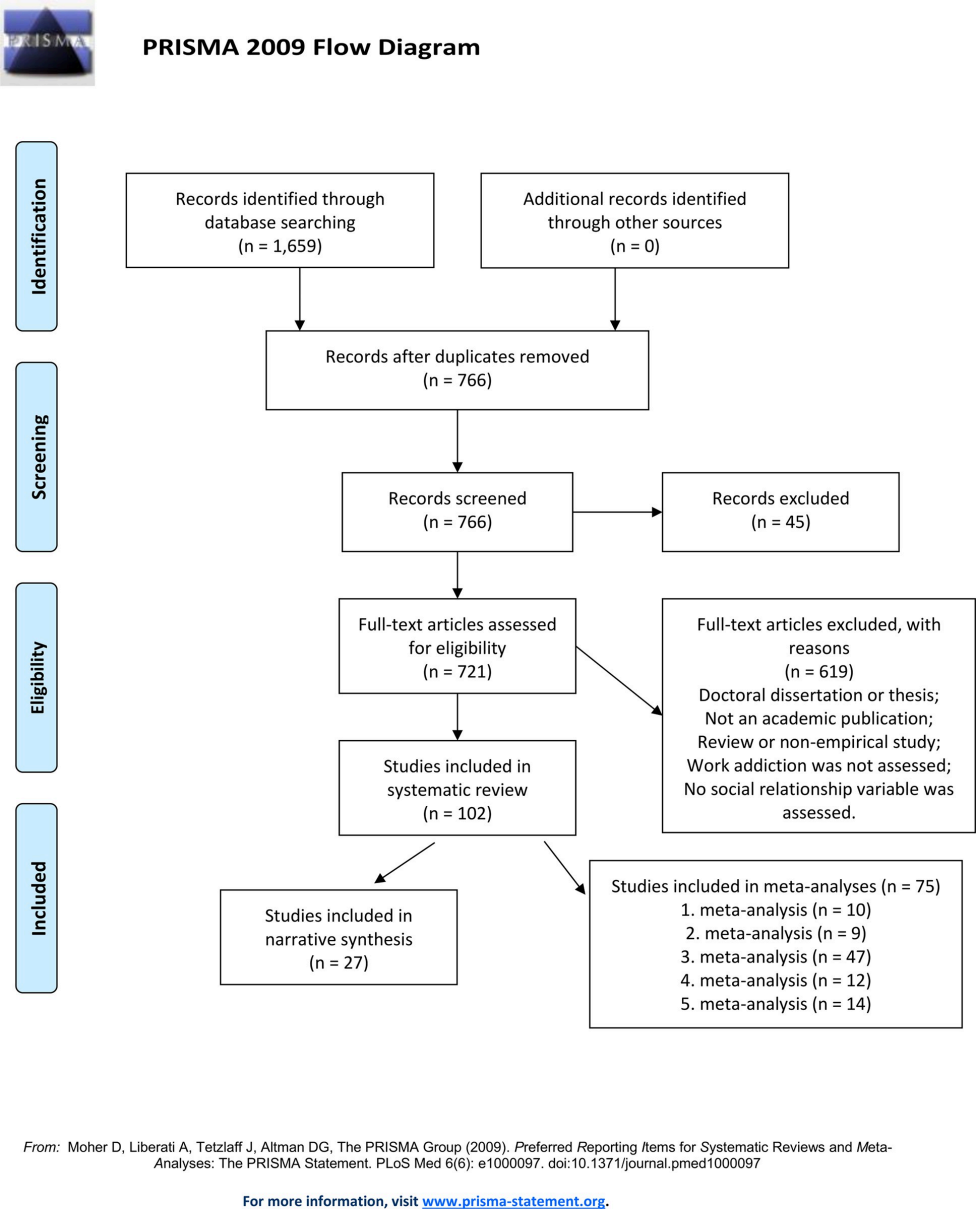
Flow diagram summarizing the screening process.

The present study was registered on the Center for Open Science’s OSF Registries page (https://doi.org/10.17605/OSF.IO/P5Z3T, Registered 5th August 2021). Structure of the systematic review followed the principles of the PRISMA-P (Preferred Reporting Items for Systematic Reviews and Meta-Analyses Protocols) [26]. PRISMA-P is an evidence-based protocol, which was developed to support authors in conducting systematic reviews and meta-analyses. Our completed PRISMA checklist is shown in S1 Appendix. Similarly, details of the search process are illustrated in the PRISMA Flow Diagram (Fig 1).

### Operational definitions

Studies concerning a wide range of focal points were included, since we aimed to examine the relationship between work addiction and different constructs regarding social relationships and social life. To analyze the vast field of social relationships in a more manageable manner, we have categorized them into six themes. This approach involves a progressively narrower focus, starting with the broader category of private life (i.e., balance between work and life), followed by general social functioning, then specific social relationships. Thus, we created six main categories for the social relationship variables, namely (1) work-life balance, (2) general social functioning, (3) actual family life, (4) family of origin and offspring of workaholics, (5) intimate relationship, and (6) other relationships i.e., relationships with friends, community, and colleagues. In S2 Appendix, we summarize the concepts that were included in the six themes.

### Search strategy

A computerized literature search was performed to collect all the relevant papers published (at any time from inception until December 2022). We conducted a systematic literature search using five databases: Web of Science, Science Direct, PsycInfo, EBSCO, and PubMed. These databases are extensively used by authors who publish systematic review and meta-analysis studies. From the perspective of work addiction research, these databases are considered the most relevant ones. While searching in the databases, we used the following keywords: “work addiction” OR “workaholism” OR “workaholic” AND “social” OR “social relationship” OR “friend” OR “family” OR “familial” OR “marriage” OR “marital” OR “married” OR “spouse” OR “spousal” OR “divorce” OR “work-life” OR “child” OR “parent” OR “parental” OR “mother” OR “father” OR “wife” OR “husband” OR “colleague” OR “supervisor”. In addition, the reference lists of all articles detected via our search were scanned for further references.

### Eligibility criteria and study selection

The study titles and the abstracts were screened for relevance. In case the abstract included information to determine eligibility, we reviewed the full text of the article. To determine which articles should be included for analysis, two of the authors independently evaluated the full-text articles. Inclusion and exclusion process is presented in detail in Fig 1. We included those quantitative studies that provided empirical data on the relationship between work addiction and any variable that assesses social relationships, or studies that investigate the relationship between two generations (i.e., parents and offspring) in the context of work addiction. Studies were included if they met the following criteria: (i) a psychometrically validated work addiction scale was used; (ii) at least one psychometrically validated scale or subscale was used to measure a social variable; (iii) parent-child relationship was investigated by a psychometrically validated scale or subscale from the perspective of work addiction; (iv) the paper was written in English; and (v) any population was involved. The articles we have included may contain population data primarily focused on adults, as work addiction is most relevant to this demographic. However, selected articles also encompass studies involving children, particularly those examining work addiction within the context of parent-child relationships. Studies were excluded, if they were: (i) theses or doctoral dissertations; (ii) non-empirical studies; (iii) non-academic publications (grey literature); (iv) reviews (e.g., theoretical studies, review articles or books reviews); (v) studies, which have not examined any relationship between work addiction and social life; and (vi) studies, which were not written in English. Two authors examined the titles and the abstracts of the identified articles, and disagreements were discussed separately, aiming for a consensus. For details of the inclusion and exclusion process, please review the PRISMA Flow Diagram Fig 1.

In addition to the systematic review, we also planned to conduct meta-analyses, which meant further selection steps. Two additional inclusion criteria were used for inclusion in the meta-analysis: (i) Correlation analysis between work addiction and the social relationship variable; (ii) Analysis of the relationship between the two variables within a single individual. The latter means that we excluded from the analysis those where the association between a person’s work addiction (e.g., supervisor, spouse) and another person’s (e.g., subordinate, partner) social characteristic (e.g., work-family conflict) was analyzed. As the topics of social relationships are extremely diverse, we decided to carry out different meta-analyses according to the previously presented six main themes. Some articles could be included in more than one meta-analysis if they covered more than one topic.

### Data extraction

Each study was evaluated according to a specific data extraction structure, which included the following data: (i) bibliographic information (author(s), year of publication and the country where the data were collected); (ii) research design (i.e., cross-sectional, longitudinal); (iii) sample characteristics (mean age, gender ratio, sample size); (iv) work addiction questionnaire (e.g., Work Addiction Risk Test [WART], Dutch Work Addiction Scale [DUWAS], etc.); (v) social relationship variables (e.g., work-life balance, marital satisfaction, etc.); (vi) measurement tool of the social relationship variable (e.g., Work-Family Conflict Scale, Kansas Marital Satisfaction Scale, etc.); (vii) Cronbach’s alpha values for all the questionnaires used; (viii) statistical methods applied (e.g.: correlation analyses, regression analyses, ANOVA, etc.); and (ix) the main results of the statistical analyses. Regarding the results, the correlation coefficients, standardized beta values, and means are given in the summary table. Where specific information was missing from the article, it was marked as not available (n/a). In the case of missing data, we attempted to contact the authors of the primary article. If our contact attempts were unsuccessful, we indicated in the table that no data were available.

Data extraction was executed separately by two authors, and we used the form shown in S3 Appendix. The form was initially tested and agreed upon in a trial run to ensure that everyone had a consistent understanding of each category. We found acceptable agreement between the authors on data extraction. Interrater reliability was high, ranging from 92.54% to 100%. Inconsistencies and disagreements were discussed and resolved via consensus. For assessing the methodological quality of the papers, we applied the Joanna Briggs Institute’s (JBI) critical appraisal tool for cross-sectional studies [27]. This appraisal is a widely recognized method for evaluating the methodology of systematic reviews and meta-analyses, which is why we chose to utilize it. This assessment was also conducted independently by two of the authors, mirroring the article review and data extraction process. Any discrepancies were resolved through separate discussions aimed at reaching a consensus. The JBI critical appraisal tool contains eight items that could be answered with “yes”, “no”, “not applicable (n/a)”, or “unclear”. We calculated a total score for each study by using one point for any affirmative answer, and zero point for any other answers. We also calculated the percentages of yes answers based on the number of available relevant criteria. The results of the risk of bias assessment can be found in S2 Table.

### Data analysis

Not all articles were included in the meta-analytical process due to not meeting all the criteria. If any of the six major themes failed to meet the meta-analysis criterion, we conducted a narrative synthesis.

The Comprehensive Meta-Analysis software was used to conduct the analyses. The dependent variable in all the meta-analyses was the correlation coefficient (r) between work addiction and a social relationship variable. In the first three meta-analyses (work-life balance, general social functioning, and family life), higher correlation coefficients indicate an association between work addiction and difficulties in social relationships. However, in the fourth and fifth meta-analyses (intimate relationships and relationships with community, friends, and colleagues), the correlation coefficient represents the association between work addiction and the quality of social relationships.

We evaluated the heterogeneity of the average effect sizes using Q-statistics and estimated I^2^. To assess publication bias, we examined the funnel plot, computed Egger’s test, and employed the Duval and Tweedie trim and fill procedure. Additionally, several moderator analyses were performed in each of the five meta-analyses. Given that many studies reported multiple effect sizes, we combined all effect sizes within each category for categorical moderators. For numerical predictors like gender ratio, mean age of the sample, and study quality, we conducted meta-regression analyses.

## Results

### Studies included in the systematic review

Fig 1 displays the PRISMA flow diagram illustrating the numbers of excluded and included studies. In total, 1,659 articles were identified and reviewed based on eligibility and exclusion criteria. As a result of checking the reference lists of all articles, no new studies (*n* = 0) were added to the existing list. After removing the duplicates (*n* = 893) with EndNote (Clarivate Analytics), we continued reviewing the abstracts of the included studies (*n* = 766). The abstract and language screening resulted in the exclusion of non-relevant records (*n* = 45). As a next step we assessed the remaining articles (*n* = 721) with full-text review. During the full-text review, we excluded studies that were doctoral dissertations, non-empirical studies (*n* = 619), or reviews and studies that did not examine any relationship between work addiction and social life (*n* = 530) or were not written in English. After completion of the entire screening process, we finalized the list of articles (*n* = 102) to be included into our systematic review (S1 Table).

### Description of the included studies

The 102 included studies were published between 1995 and 2022 (until December 31). Nine studies (8.8%) were published between 1995 and 2000, 19 studies (18.6%) between 2001 and 2010, and almost three quarters of the papers (N = 74; 72,5%) were published between 2011 and 2022. More than one-third of the studies (n = 38) were conducted in Europe and one-third were carried out in North America (*n* = 35). The remaining studies were conducted in Asia (*n* = 14), Australia (*n* = 5), Middle East countries (*n* = 7), and South America (*n* = 3). A convenience sample was used in all studies. A total of 15 articles applied longitudinal research design, and all the other studies (*n* = 87) used cross-sectional design. Most of the studies (*n* = 99) involved adult participants, and only three studies investigated both parents and their children. The mean age of the adult samples was between 22.14 and 54.7 years. The largest sample included 8,419 participants, and the smallest sample involved 24 individuals. Altogether, the total number of adult participants included in the current systematic review was 66,186, and a total of 333 families were included. The average ratio of males in the studies was 40.37%.

As mentioned above, the included quantitative studies used at least one psychometrically validated work addiction scale. S1 Fig presents all thirteen different work addiction measures applied in the studies and the number of the studies using these instruments.

The quality analysis of the primary studies (S2 Table) showed that the average quality rating of 102 included studies was 88%. About half of the studies (*n* = 56; 55%) reached the maximum scores (100%) in the checklist, and more than one-fifth of the studies (*n* = 20; 19.6%) were rated 86%. A total of 14 studies (13.7%) were rated 71.4% on the checklist, and nine of them (8.8%) reached 57.1%. Finally, only three studies (2.9%) did not reach the 50% level of the quality rating, these articles scored 43%.

### Meta-analyses

A total of 22 articles were excluded from the meta-analyses due to a lack of correlation analysis, and a further 5 articles were excluded due to the analysis was conducted between a person’s work addiction and another person’s social characteristics. The excluded studies comprised 6 articles on general social functioning, 9 articles on actual family life, 7 on family of origin and offspring of workaholics, 4 on intimate relationships, and 2 on relationships with friends, community, and colleagues.

A total of 75 articles were included in the meta-analytic part of the study. Although six themes were initially identified, and theoretically, six meta-analyses could have been conducted, one of the themes lacked sufficient eligible studies. Consequently, we were able to conduct five meta-analyses. For each meta-analysis, we have included the following number of articles: (1) Work-life balance: 10 studies; (2) General social functioning: 9 studies; (3) Current family life: 47 studies; (4) Intimate relationships: 12 studies; and (5) Community, friendship, and colleagues: 14 studies. Regarding the topic of ‘family of origin and offspring of workaholics’, there were very few studies available, none of which met the inclusion criteria for the meta-analysis. Therefore, we processed the studies on this topic in the form of a narrative synthesis, and the results are presented subsequent to the five meta-analyses.

#### 1. Meta-analysis: Work addiction and work-life imbalance

**Fig 2:**
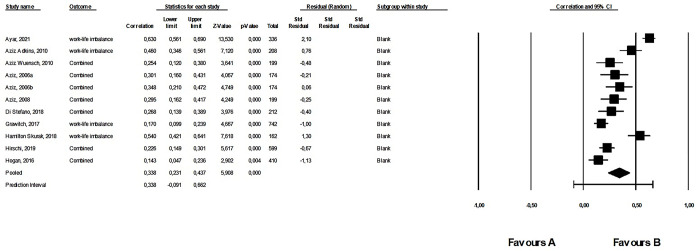
Forest plot of the first meta-analysis: The relationship between work addiction and work-life imbalance.

Results of 11 studies, including data from 3,415 participants, were synthesized. There were no outliers. The average correlation between work addiction and work-life imbalance was positive, moderate, and significant (*r* = .338, 95% CI: 0.231; 0.437, *p* < .001). This was a heterogeneous effect (see S3 Table). The forest plot for the second meta-analysis is shown in Fig 2. Regarding publication bias, Egger’s test was not significant (intercept: 5.48, *p* = .148), and the funnel plot showed asymmetry (see S2 Fig), however, Duval and Tweedie’s Trim and Fill calculation showed a smaller but significant average effect size (*r* = .213, 95% CI: 0.085; 0.335). According to Rosenthal’s classic fail-safe *n* of 995, it was a robust effect.

We could analyze the effects of two social relationship variables separately. While work-life enrichment (based on only 1 study) does not show a significant relationship with work addiction (*r* = –.045, CI: –0.125; 0.035, *k* = 1, Z = –1.11, *p* = 0.268), work-life imbalance exhibits a medium and significant positive relationship with work addiction (*r* = .362, 95% CI: 0.251; 0.463, *k* = 11, Z = 6.08, *p* < .001).

#### 2. Meta-analysis: Work addiction and difficulties in general social functioning

**Fig 3:**
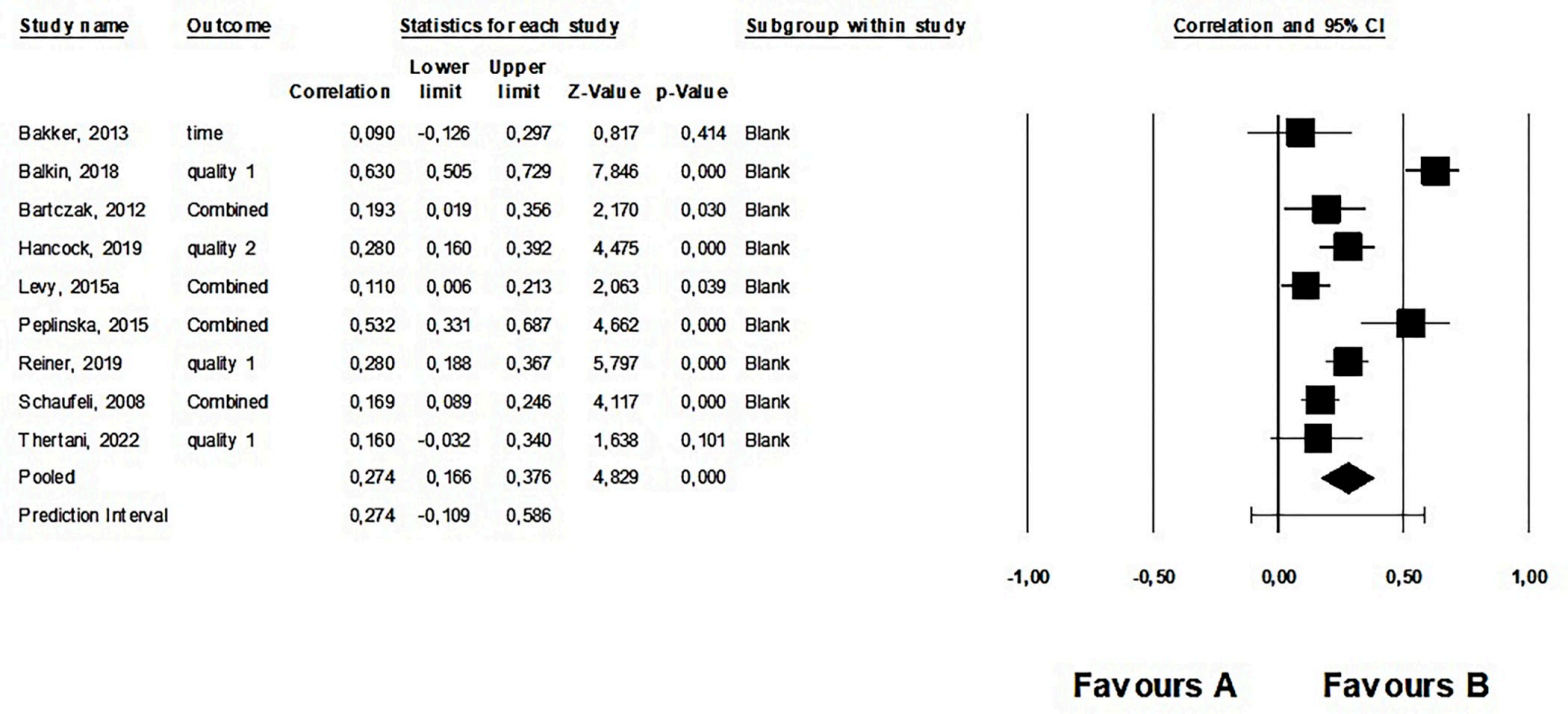
Forest plot of the second meta-analysis: The relationship between work addiction and difficulties in general social functioning.

Results of 9 studies, including data from 2,088 participants, were synthesized. There were no outliers. The average correlation between work addiction and difficulties in general social functioning was small but positive and significant (*r* = .274, 95% CI: 0.166; 0.376, *p* < .001). This was a heterogeneous effect (see S3 Table). The forest plot for the third meta-analysis is shown in Fig 3. Regarding publication bias, the funnel plot was symmetrical (see S3 Fig) but the Egger’s test was not significant (intercept: 2.79, *p* = .249). However, Duval and Tweedie’s Trim and Fill calculation showed a significant average effect size (*r* = .274, 95% CI: 0.165; 0.376). According to Rosenthal’s classic fail-safe *n* of 285, it was a robust effect.

When we analyzed each of the sub-themes within the social functions, we found that work addiction exhibited a significant moderate negative relationship with the quality of social life and a significant positive but weak relationship with social dysfunction and the amount of social conflict (Table 1). However, no significant relationship with the amount of time spent with others and the amount of social support was found for work addiction (Table 1).

**Table 1:** Results of the moderator analyses focusing on the social variables in context of general social life.

Variable	*k*	*n*	*r*	95% CI	*Z*	*p*
**Quality of social life**	4	875	**–.349**	–0.510, –0.165	–3.611	< .001
**Social dysfunction**	2	713	**.191**	0.119, 0.261	5.152	< .001
**Lack of support**	2	415	.326	–0.133, 0.669	1.405	.160
**Conflicts with others**	1	587	**.146**	0.066, 0.225	3.562	< .001
**Time spent with others**	1	85	.090	–0.126, 0.297	0.817	.414

*Note*. Significant correlations in bold.

#### 3. Meta-analysis: Work addiction and difficulties in family relationships

**Fig 4:**
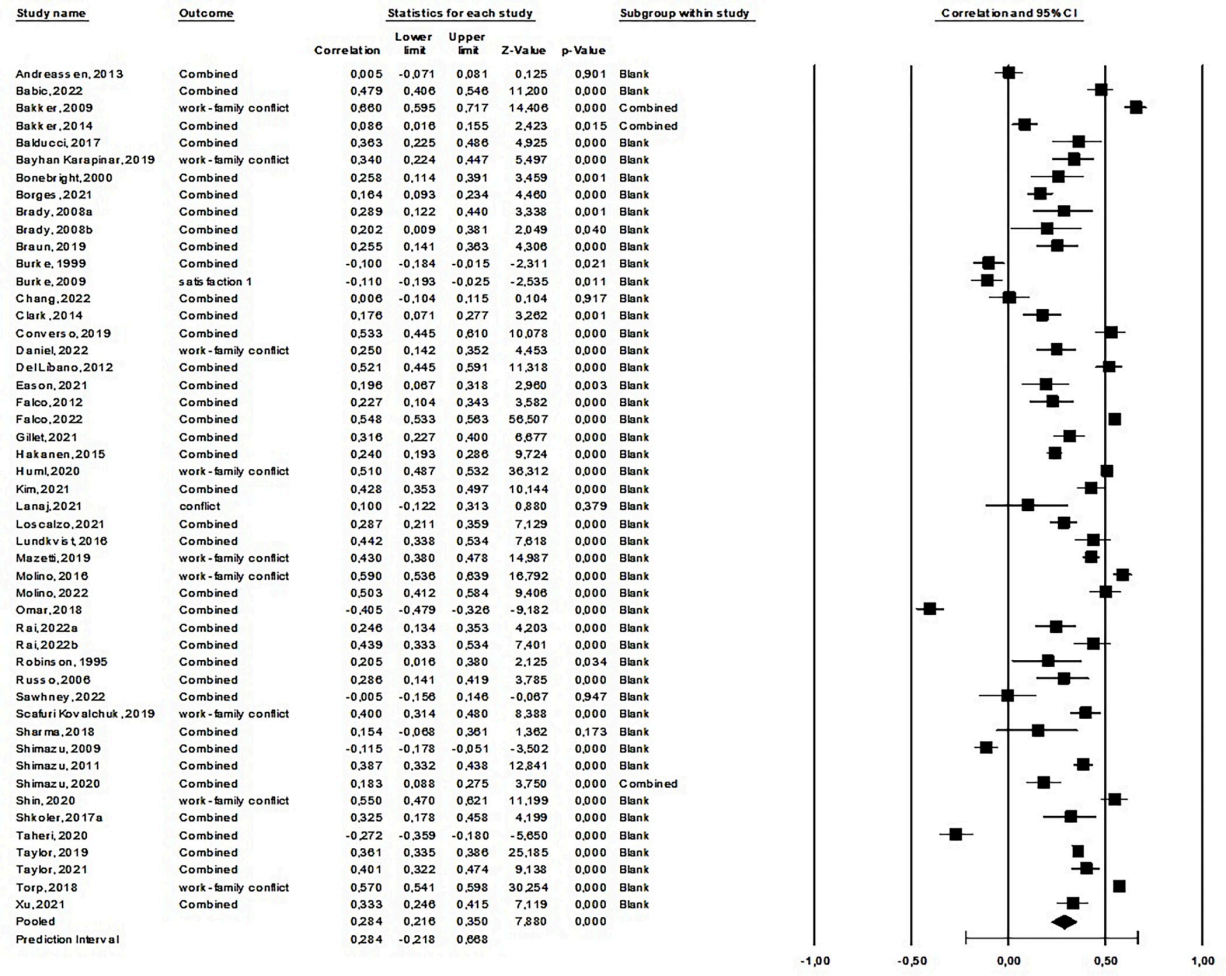
Forest plot of the third meta-analysis: The relationship between work addiction and difficulties in family life.

After excluding an outlier [28], we synthesized 49 studies reporting on 37,917 participants in the first meta-analysis. The average correlation between work addiction and difficulties in family-related social relationships was small but positive and significant (*r* = .284, 95% CI: 0.216; 0.350, *p* < 001). This effect was heterogeneous (see S3 Table). The forest plot for the first meta-analysis is shown in Fig 4. Regarding publication bias, Egger’s test was significant (intercept: –5.78, *p* < .001), the funnel plot was symmetrical (see S4 Fig), and Duval and Tweedie’s Trim and Fill calculation showed a significant average effect size (*r* = .284, 95% CI: 0.216; 0.349). Finally, according to Rosenthal’s classic fail-safe *n* of 6,719, the effect appeared robust.

Analyses were also carried out separately for each of the main social relationship variables. The largest number of studies was on work-family conflict (*k* = 42), showing a significant positive relationship of moderate strength (Table 2). There is also a significant positive but weak relationship between work addiction and family dysfunction, but only one study supports this finding. A significant, yet weak, negative relationship is found with work-family facilitation, satisfaction with family relationships, and family engagement. Although based on only one study, a significant, moderate-strength, negative relationship is observed between work addiction and family support. However, work addiction shows no relationship with family conflict or the quality of family relationships (Table 2).

**Table 2:** Results of the moderator analyses focusing on the social variables in context of family life.

Variable	*k*	*n*	*r*	95% CI	*Z*	*p*
**Work-family conflict**	42	30,792	**.412**	0.374, 0.449	19.080	< .001
**Work-family facilitation**	8	5,395	**–.160**	–0.24, 0.078	–3.773	.001
**Satisfaction**	7	3,380	**–.113**	–0.146, –0.079	–6.551	< .001
**Dysfunction**	1	107	**.271**	0.085, 0.438	0.2834	.005
**Conflict in the family**	1	80	.100	–0.122, 0.313	0.88	.379
**Family engagement**	1	169	**–.175**	–0.318, –0.032	–2.279	.023
**Quality of relationships**	1	107	–.357	–0.223, 0.156	–0.035	.721
**Support**	1	322	**–.370**	–0.461, –0.272	6.937	< .001

*Note*. Significant correlations in bold.

#### 4. Meta-analysis: Work addiction and the quality of intimate relationship

**Fig 5:**
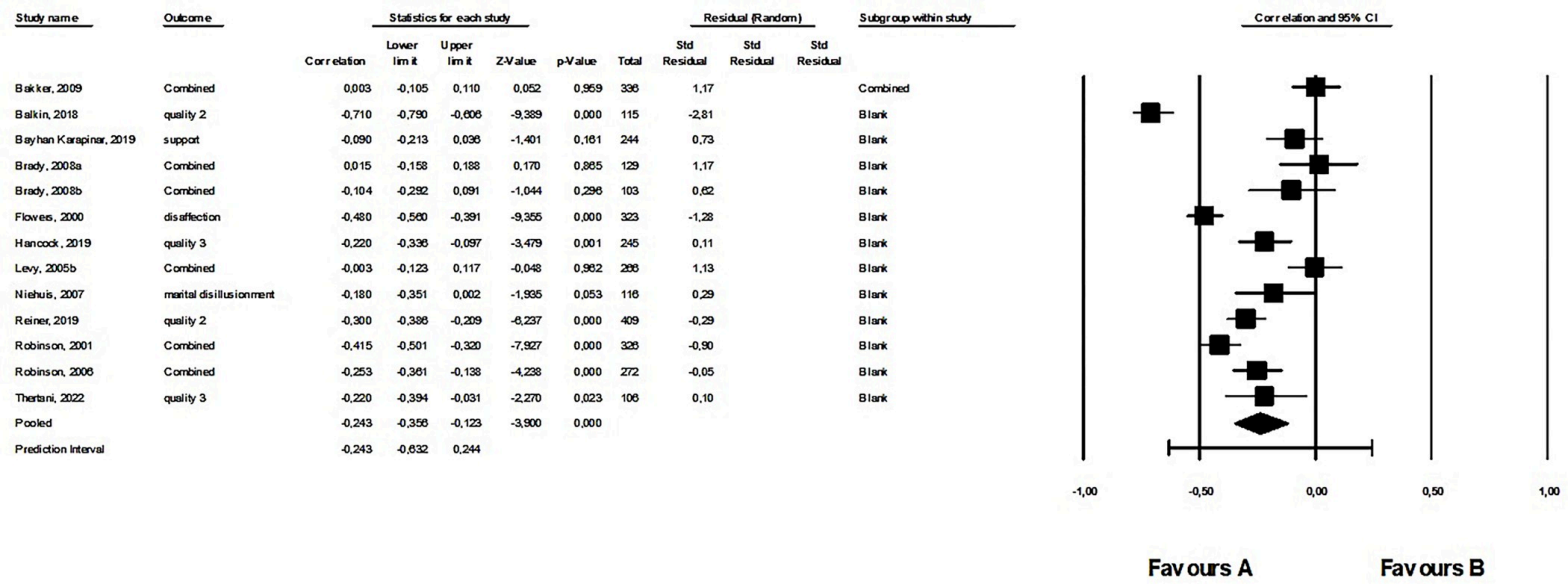
Forest plot of the fourth meta-analysis: The relationship between work addiction and the quality of intimate relationships.

Results of 13 studies, including data from 2,990 participants, were synthesized. There were no outliers. The average correlation between work addiction and the quality of intimate relationship was small but negative and significant (*r* = –.243, 95% CI: –0.356; –0.123, *p* < .001). This was a heterogeneous effect (see S3 Table). The forest plot for the third meta-analysis is shown in Fig 5. Regarding publication bias, Egger’s test was not significant (intercept: 2.94, *p* = .945) and the funnel plot showed asymmetry (see S5 Fig), however, Duval and Tweedie’s Trim and Fill calculation showed a significant average effect size (*r* = –.315, 95% CI: –0.427; –0.193). According to Rosenthal’s classic fail-safe *n* of 565, it was a robust effect.

When analyzed separately for each theme, there is a significant negative relationship of moderate strength between work addiction and relationship quality and positive feelings in the relationship (Table 3). There is also a moderate but positive relationship between work addiction and relationship disaffection. However, there is no significant correlation between relationship satisfaction, relationship support, and work addiction (Table 3).

**Table 3:** Results of the moderator analyses focusing on the social variables in context of the quality of intimate relationships.

Variable	*k*	*n*	*r*	95% CI	*Z*	*p*
**Quality of relationship**	4	875	**–.385**	–0.579, –0.150	–3.120	.002
**Satisfaction with relationship**	4	834	–.021	–0.090, 0.047	–0.615	.539
**Disillusionment**	3	714	**.253**	0.152, 0.427	3.940	< .001
**Support**	2	580	–.025	–0.142, 0.093	–0.416	.677
**Disaffection**	1	323	**.480**	0.391, 0.560	9.355	< .001
**Positive feelings**	1	326	**–.415**	–0.501, –0.321	–7.947	< .001

*Note*. Significant correlations in bold.

#### 5. Meta-analysis: Work addiction and the quality of relationships with friends, community, and colleagues

**Fig 6:**
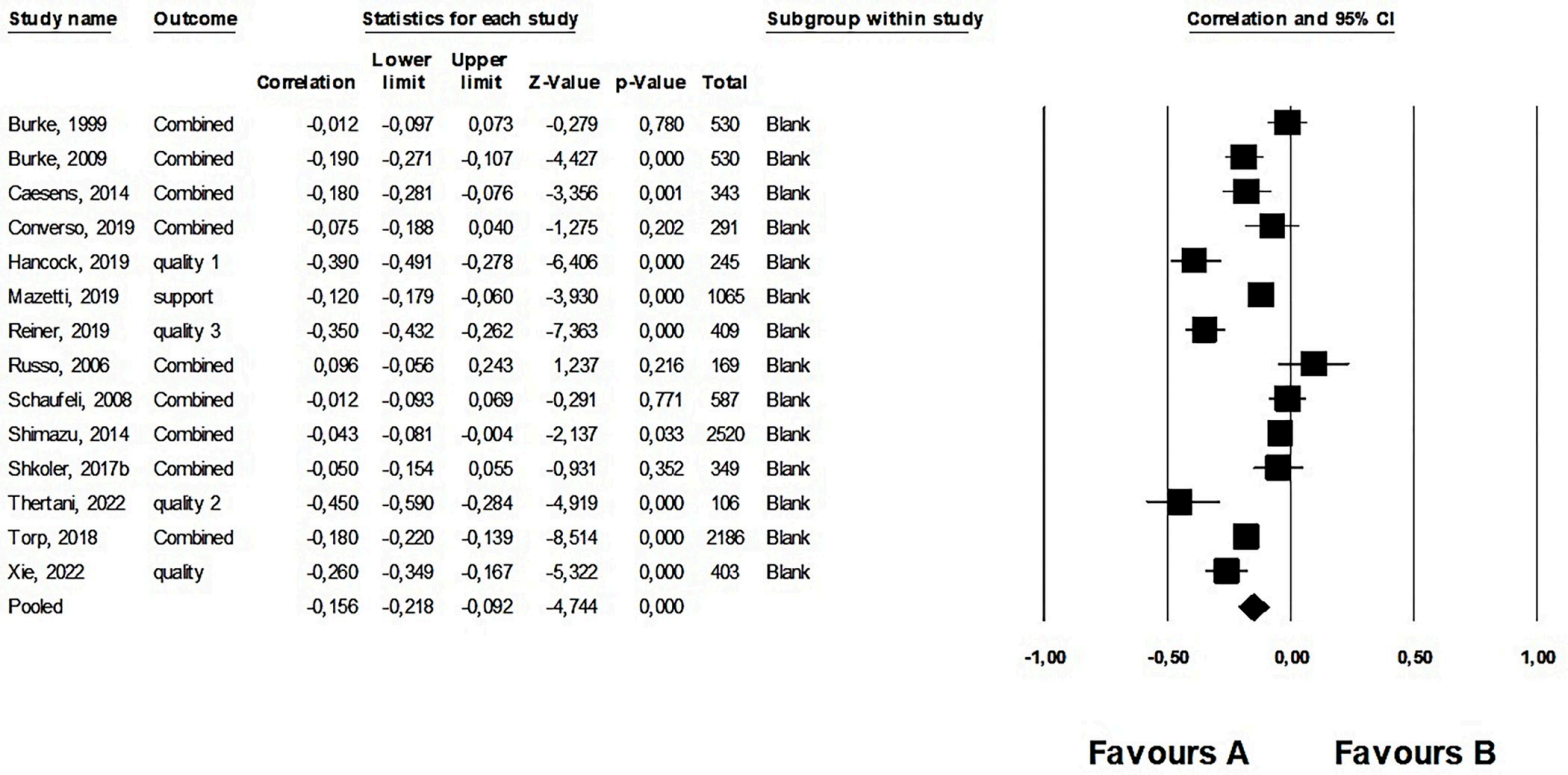
Forest plot of the fifth meta-analysis: The relationship between work addiction and quality of relationships with friends, community, and colleagues.

After excluding an outlier [29], we synthesized 14 studies reporting on 9,733 participants. The average correlation between work addiction and the quality of relationships with friends, community, and colleagues was small but negative and significant (*r* = –.156, 95% CI: –0.218; –0.092, *p* < .001). This was a heterogeneous effect (see S3 Table). The forest plot for the first meta-analysis is shown in Fig 6. Regarding publication bias, the funnel plot was symmetrical (see S6 Fig) but Egger’s test was not significant (intercept: –2.10, *p* = .275). However, Duval and Tweedie’s Trim and Fill calculation showed a significant average effect size (*r* = –.155, 95% CI: –0.218; –0.091). According to Rosenthal’s classic fail-safe *n* of 584, it was a robust effect.

By analyzing each of the social variables separately, we find that while relationship quality and relationship cohesion show a weak but significant negative relationship with work addiction, social support, relationship satisfaction, and conflict levels are not related to work addiction (Table 4).

**Table 4:** Results of the moderator analyses focusing on the social variables in context of the quality of relationships with friends, community, and colleagues.

Variable	*k*	*n*	*r*	95% CI	*Z*	*p*
**Quality**	5	1,512	**–.298**	–0.422, –0.163	–4.220	< .001
**Support**	4	3,619	–.044	–0.122, 0.035	–1.089	.276
**Satisfaction**	2	1,060	–.102	–0.273, 0.074	–1.135	.256
**Cohesion**	1	2,186	**–.205**	–0.245, –0.164	–9.716	< .001
**Conflict**	1	291	.075	0.040, 0.148	1.275	.202

*Note*. Significant correlations in bold.

Since this meta-analysis included several types of non-family-related social relationship factors, moderator analysis was also conducted for each type of relationship. Again, all relationships remained significantly negative, and although all were weakly correlated, with varying strengths. Thus, the strongest correlation was found between work addiction and community. Additionally, within the workplace, the negative correlation with coworkers was stronger than with the manager (Table 5).

**Table 5:** Results of the moderator analyses focusing on the social relationship categories in context of the quality of relationships with friends, community, and colleagues.

Relationship	*k*	*n*	*r*	95% CI	*Z*	*p*
**Workplace**	9	7,913	**–.093**	–0.160, –0.025	–2.674	.007
**Manager**	7	5,102	**–.089**	–0.165, –0.012	–2.253	.024
**Coworker**	5	5,927	**–.113**	–0.213, –0.011	–2.169	.030
**Friends**	3	3,580	**–.079**	–0.134, –0.024	–2.800	.005
**Community**	5	1,820	**–.286**	–0.431, –0.127	–3.460	.001

*Note*. Significant correlations in bold.

Furthermore, a moderator analysis was conducted for all five meta-analyses on the work addiction scales. The results of this analysis are summarized in S4 Appendix.

#### Meta-regression analyses

The mean age of the sample served as a significant moderator between work addiction and work-life imbalance, however, the coefficient was very small (Coefficient = –.023; Z = –2.76; *p* = .005). This result indicates that among younger individuals, the relationship between work addiction and work-life imbalance is higher. Mean age did not serve as a significant moderator in the other four meta-analyses (see Table 6). Concerning the gender ratio of the samples, we observed a significant moderator effect only for work-life imbalance. We found a very small negative effect (Coefficient = –.01; Z = –8.10; *p* < .001), suggesting that the positive relationship between work addiction and work-life imbalance is stronger among females than males. In the other four meta-analyses, we did not observe any significant moderator effect of the gender ratio (Table 6). Based on the quality scores of the studies, significant effects were found in only one of the five meta-analyses. There exists a positive effect regarding difficulties in family relationships; specifically, higher-quality studies indicate a stronger association between work addiction and challenges in family relationships. It is essential to note, however, that the effect size in this context is very modest (Coefficient = .006; Z = 2.36; p = .018) (Table 6).

**Table 6:** Results of the meta-regression analyses.

Moderator	Outcome	Coefficient	*Z*	*p*
**Mean age**	**Family life**	0.005	0.61	.540
	**Work-life imbalance**	**–0.023**	–2.76	.005
	**General social life**	–0.018	–0.66	.506
	**Intimate relationship**	–0.002	–0.12	.903
	**Friends, community, colleagues**	–0.003	–0.46	.646
**Gender**	**Family life**	0.005	0.22	.827
	**Work-life imbalance**	**–0.010**	–8.10	< .001
	**General social life**	0.001	0.41	.685
	**Intimate relationship**	0.001	0.27	.787
	**Friends, community, colleagues**	0.002	1.04	.299
**Quality of the articles**	**Family life**	**0.006**	2.36	.018
	**Work-life imbalance**	0.005	1.38	.168
	**General social life**	0.002	0.28	.778
	**Intimate relationship**	0.004	0.67	.501
	**Friends, community, colleagues**	0.003	1.38	.167

*Note*. Significant effect sizes in bold.

#### Narrative synthesis of the sixth main topic: Family of origin and offspring of workaholics

Since the included studies did not meet the inclusion criteria for meta-analyses, a narrative synthesis of the articles on this topic was performed. Only a few studies (*n* = 9) have examined the association between work addiction and social relationships focusing on two generations, namely parents and offspring. In two studies, participants were asked about both their symptoms of work addiction and their perceptions about their parents and family of origin. On the one hand, the *perceived level of health in the original family* did not show significant correlation with current work addiction [30]. Namely, neither the intimacy, nor the autonomy in the family of origin was related to future work addiction of the offspring. On the other hand, *parental work addiction*, as rated by the offspring, showed a significant, positive, but weak correlation with the work addiction of the adult child [31, 32] and it was true for both mothers and fathers of the offspring. Regarding the contradictory results, it is noteworthy that the former study [30] presents methodological concerns based on our quality analysis, scoring only 43% (see S2 Table). This raises questions about the reliability and robustness of its findings.

A total of six studies explored the relationship between parental work addiction and the child’s personality, psychological health, and the relationship with the parent. In a longitudinal study involving only mothers and their infant daughters, maternal workaholic personality (applying the SNAP questionnaire) significantly predicted *shared positive affective ambience* between mothers and children [33]. Further, in a cross-sectional study asking parents and their children (*M*_age_ = 10.6), the correlations between parental work addiction and the *child’s self-esteem*, *locus of control*, *and anxiety* were not significant [34]. Based on the latter two studies, parental work addiction does not have a negative effect on the child’s psychological health. However, it is advisable to approach the results of the latter study with caution, given its lower quality (see S2 Table). In contrast, four studies found controversial results, especially regarding fathers. Robinson and Carroll [35] found a significant, positive, and strong correlation between work addiction of the parents (rated by the offspring) and the *parentification of the child*. In a further study, significant differences were found comparing the personality and well-being of children of workaholic parents and non-workaholic parents [36]. While no significant difference was found between the *self-concept* scores of children of workaholic and non-workaholic parents, children of workaholics had higher *depression* scores than children of non-workaholics. Moreover, regarding depression, *external locus of control*, *and anxiety* of the child, the differences were significant only among fathers but not among mothers. It was found that children of workaholic fathers had higher level of depression, anxiety, and external locus of control. This gender difference was found in two further studies too. In a recent Japanese study [37], the father’s work addiction predicted the *child’s emotional and behavioral problems*, however, this effect was not significant for mothers. Finally, the adult offspring’s work addiction was predicted by only the father’s work addiction (Working Excessively) but not by the mother’s working behavior [38].

## Discussion

Several theoretical models of work addiction suggest that excessive and compulsive overwork associates with problems in social relationships [6, 7, 14, 15, 22, 39]. While some models are based on empirical findings of work addiction, others are built only on clinical observations or anecdotal information. The present study aimed to systematically review and meta-analyze all the available empirical, quantitative studies that investigated the associations between work addiction and social relationships. To the best of the authors’ knowledge, this is the first meta-analysis focusing on this specific topic. By applying five databases and electronic sources, we included a total of 102 studies to our systematic review. From these, we were able to analyze 75 relevant studies in five meta-analyses.

The term ‘work-life balance’ comprises the harmony between the individual’s work-related and private [i.e., nonwork life and roles], therefore, it highly associates with the person’s social relationships. Our first meta-analysis shows that work addiction has a significant and moderately positive correlation with work-life imbalance. These findings serve as evidence for the “salience” component of work addiction [15]: addictive workers neglect other areas of their life [39] and cannot properly fulfill other roles and obligations. While work engagement is characterized by a positive spillover effect between work and nonwork areas [39], we still do not have enough knowledge on work addiction in the context of work-life enrichment. Based on our meta-analysis, there appears to be no significant relationship between work addiction and work-life enrichment. However, it’s important to note the limited data available, with only one study in this area. Further exploration in future research is warranted.

Regarding empirical studies on the associations between work addiction and general social functioning, we found that although a small amount of research has been conducted in this field, most of the studies reported congruent findings. A higher level of work addiction correlates with lower general social functioning. Individuals who report higher levels of work addiction tend to exhibit lower quality in their social lives and score higher on scales measuring social dysfunction and social conflict. However, work addiction does not appear to be associated with the amount of time spent with others or the level of social support received. Due to very limited longitudinal research in this area, we were unable to incorporate this variable into our meta-analyses. A longitudinal design would offer greater insight into whether a causal relationship exists between social dysfunction and work addiction. It is conceivable that difficulties in social functioning might lead the person to find other activities in life, e.g., invest more time in work than in nonwork activities. This assumption reminds us of the predictive role of escape motives in different addictions (e.g., alcohol use disorder, gaming disorder) [40, 41]. Escape motivation refers to the mood modification component of addictive disorders [17] describing that the person regularly uses the specific behavior (or substance) for emotion regulation or mood management purposes. Regarding work addiction, more studies are needed to explore the possible role of escapism motivation in overwork and its relation to social functioning.

Based on our third meta-analysis, it was found that work addiction has a significant and weak positive correlation with difficulties in actual family relationships. Our analysis showed that the strongest positive association has been found between work addiction and work-family conflict (WFC). In other words, it means that work addiction involves significant conflicts between work roles and family roles, as an earlier review involving only 18 studies also found [42]. This clear association is also evidence for the maladaptive pattern of work addiction, as many theories suggest. Especially, the component model of addiction [17] emphasizes that several (i.e., intrapsychic and interpersonal) conflicts arise from the person’s obsessive and addictive behavior. This causal relationship (i.e., from addictive work to family conflicts) has been supported by longitudinal studies [43] showing that an earlier work addiction predicted future WFC. Interestingly and importantly, the association has not been confirmed from the other direction: the level of WFC has not predicted work addiction [44]. It suggests that the obsessive pattern of work increases the risk of conflict in family life, however, more conflicts between work and family is not a risk factor of being an addictive worker.

There are several theories that explain the elevated level of WFC in work addiction [45]. Since workaholics overcommit themselves with tasks and spend an enormous time with work, their working activities regularly flow into their family life. They might have difficulties drawing boundaries between work and family and separating these areas from each other (i.e., not working at home, not ruminating on working tasks at home, etc.). In other words, if addictive workers are ‘integrators’ who have very thin boundaries between work and family [46], they might experience more conflict arising from working at home [47]. We can also interpret the association between work addiction and WFC by crossover and spillover effects [48]. Since addictive workers experience an elevated level of stress [49], they often have to face the problem that work-related stress spills over in family roles and crosses over to the individual’s family members (e.g., partner, parents, and children). Finally, the theory of the conservation of resources [50] can also be used to explain WFC in work addiction. If a person expends resources heavily in one specific area (i.e., work), it depletes resources available for other areas (i.e., family life). When the individual becomes exhausted by overwork, they may lack the capacity to engage, assist, or even spend time with family members, leading to conflicts between them.

When examining positive indicators of actual family social life, our meta-analysis highlights two significant findings. Firstly, there is limited research available in this area, with only one or two studies identified. Secondly, the results indicate a negative association between work addiction and positive family factors, including work-family facilitation, satisfaction with family relationships, and family engagement. These finding suggest that a person who obsessively and excessively works every day to achieve irrationally high goals [51] or because of high demands came from the workplace [52] does not have enough capacity to enrich and develop his/her family relationships. It is also possible that the correlation is attributable to the high expectations of workaholics towards their significant others [53], along with their narcissistic traits [54]. These traits might lead them to expect greater support and positive emotions from their family members, in addition to fulfilling their demanding work commitments. When these expectations are not met, it can lead to conflicts in general. Overall, our results suggest that work addiction is associated with more family relationship difficulties. These findings are in line with the studies that reported dysfunctional family mechanisms in other addictions: alcohol use disorder, substance use disorder, or gambling disorder [55, 56]. It seems that work addiction shows similarities with other addictive disorders in this respect as well.

Our fourth meta-analysis reveals that work addiction is also linked to the quality of intimate relationships: higher levels of work addiction correlate with poorer relationship quality. It is crucial to note that there has been limited empirical research in this domain thus far. However, our moderator analyses indicate that the quality of relationships is moderately associated with work addiction. Individuals exhibiting stronger symptoms of work addiction are more likely to experience disillusionment, disaffection, and a lack of positive emotions within their relationships. Interestingly, satisfaction with the relationship and support between the partners are not correlated with work addiction. This observation may suggest that workaholics may indeed have partners, and while they may provide mutual assistance and support, emotional fulfillment remains a primary concern. These empirical results are congruent with observations from family therapy [20], namely, the partner of the workaholic person feels oneself in the second position behind work, being neglected, unloved, and not respected by the addicted worker. There are several possible explanations for the problematic intimate relationship of addictive workers. First, work overload might drain the workaholics’ resources so much that they have no energy left for their intimate relationships [57]. A second possible explanation indicates that an unhappier, more problematic intimate relationship might facilitate the person to spend more time with work and to get more reinforcements in workplace than they get in their intimate relationship. Again, it is not possible to answer this question due to the dominance of cross-sectional designed studies, however, there are earlier theories suggesting that work addiction is an attempt to escape from unpleasant experiences through excessive work [45]. For instance, Minirth et al. [58] stated that individuals with work addiction use overwork to avoid getting touch with emotions and intimacy.

Although most of the studies on the association between work addiction and social relationships focused on family relations, the present review and meta-analysis has also included those research that investigated other relationships (community, friends, and colleagues). In our fifth meta-analysis, we discovered a weak but negative correlation between work addiction and relationship quality across community, friends, and coworker relationships. These correlations are particularly noticeable within the community and among colleagues. Furthermore, while significant negative correlations were observed between work addiction and relationship quality as well as relationship cohesion, there was no association found with relationship satisfaction or conflict levels. Our findings underscore another downside of work addiction: the person feels that there are problems not only in her/his family but also in friendships and other relationships. It seems that work addiction is not as supported in the immediate environment as it would be expected. As many myths suggest, work addiction is seen as a “positive addiction” that is supported by the society [5, 7, 59]. Although workaholics can be respected by other people because of their persistence and diligence [60], they report fewer positive experiences from their social environment than others, as our review found. Moreover, work addiction is associated with more problematic social life even in the workplace. Although very little research has been conducted on this topic, those people who have higher work addiction scores report poorer workplace support and team cohesion than people who are less affected by work addiction. These results might be explained by some personality characteristics of workaholics: these people show higher level of type A personality [61], narcissism [62], other-oriented perfectionism [63], and obsessiveness [64] that make cooperation with them much more difficult than with others. Moreover, work addiction positively relates to competitive organizational climate [65], therefore, less supportive workplace environment can also be an antecedent of the problem. Interestingly, individuals affected in work addiction have more conflicts with their leaders and receive less support from them too. It would be expected that leaders are pleased with the employee’s overcommitment and overworks, but the studies show conflicting and less supportive relationship between workaholics and their supervisors. Leaders might also face the same problems with workaholics as their colleagues do. In addition, conflicting and non-cooperative workplace relationships associate with further problems in the workplace. Empirical studies found that work addiction is associated with uncivil workplace behaviors [66], burnout [25], and more intensive quit intentions [67]. Due to these correlates of work addiction, it is clearly not beneficial for superiors to have a work-addicted employee.

The number of available studies and the heterogeneous methodology did not permit the execution of the planned sixth meta-analysis; therefore, a narrative synthesis was conducted for the sixth topic. We reviewed studies that investigated the correlation between work addiction and the quality of family relationships involving multiple generations. On the one hand, there is not enough evidence to describe the family of origin of workaholics. As a current qualitative study showed [68], addicted workers reported a higher level of parental workaholism than non-addicted workers, however, perceived level of family health did not have connection with later work addiction. On the other hand, based on four studies, workaholic males experience more problems regarding their children’s health than non-workaholics: paternal work addiction is related to higher emotional and behavioral problems of the child and higher later adult workaholism too. Again, defining the causal relationships is very difficult, however, some authors suggest that the child’s mental problems can be a result of parental work addiction [7, 60]. When considering the implications of work addiction, it becomes crucial to differentiate between the short-term advantages, such as attaining higher positions and salary, and the long-term costs, particularly pertaining to the health issues faced by offspring [13]. Again, it is worth paying attention to the similarities with other addictions, such as children of alcoholics who have several psychological problems in their childhood and adulthood because of the parental addictive disorder [69, 70].

Our meta-analysis also revealed that work addiction and poorer social relationship functioning remain consistent across gender and age groups. Across the five meta-analyses, significant effects were only found for work-life balance, but these effects were so minimal that they can be considered inconsequential. Collectively, these findings indicate that work addiction and various social relationship challenges impact individuals of both sexes similarly, with no discernible differences across age groups. Finally, it is important to note that in all five meta-analyses, no significant publication bias was detected, enhancing the reliability of our results. Concerning the quality of publications, a significant effect was observed in only one of the five meta-analyses, but it was minimal and therefore deemed negligible. This indicates that methodological disparities among the studies and their overall quality have minimal influence on the obtained results.

## Limitations and future directions

Limitations should also be considered when interpreting the results. First, there is a large variability in the number of studies regarding specific topics. In several cases, only a very limited number of (i.e., one or two) studies have been published. Therefore, the results can only be considered with caution. Second, the vast majority of the included studies were based on a cross-sectional design. It means that although we have quite extensive knowledge on social life in work addiction, the causal relationships between these factors are still unclear. Third, the studies were conducted using a broad spectrum of samples, all of which employed convenience sampling. Consequently, caution must be exercised when generalizing the results across diverse populations. Fourth, most of the studies examined only the perspective of one person (i.e., the individuals affected by work addiction), and the experiences or ratings of important others (e.g., partners, family members, colleagues, friends) were not explored. Therefore, self-rating biases should also be taken into account when interpreting the results. Fifth, distortions by self-report measure can be stronger among workaholics because of their personality characteristics: several studies showed that work addiction correlates with low global self-esteem and high negative affectivity [71]. It is possible that people with a higher level of work addiction evaluate their social relationships less favorably as well as themselves. Lastly, it is worth noting that a limitation of our study was the inability to conduct a meta-analysis on one of the six topics due to non-compliance with the inclusion criteria, leading us to perform a narrative synthesis instead. While narrative synthesis is less methodologically rigorous compared to a meta-analysis, it was necessary given the circumstances. We hope that future empirical studies on this topic will provide enough data to facilitate a meta-analysis.

Based on the present comprehensive overview of this field, we would suggest the following future research directions: 1) Although it is clear that work addiction is associated with poorer quality of social relationships, the origins of these difficulties should be investigated. For instance, the *attachment style* and *early social experiences* of workaholics should be comprehensively studied. 2) We can understand the negative characteristics of social relationships in work addiction more deeply if we know more about the individuals’ *social and emotional competencies*. It would be fruitful to have a deeper knowledge on the role of emotion regulation mechanisms, emotional intelligence, and social skills in work addiction. This would also be relevant because several studies already showed that different forms of addictive disorders (both chemical and behavioral addictions) are associated with deficits in emotion regulation and emotional intelligence [72, 73]. 3) As we mentioned earlier, excessive and obsessive work may serve as a coping strategy against negative experiences and affective states, and thus, those involved may escape from emotional and social difficulties to excessive work. Future studies should investigate the predicting role of *escape motivation* in work addiction because it has never been investigated. 4) Although it was found that individuals affected with work addiction report more problems in their intimate relationship, marriage, and family life, separation and divorce are not more documented among them. It would be beneficial to explore the factors that can explain why those people who have problems in their intimate relationship still stay together. As in other addictions (e.g., alcoholism, substance use disorder), it would be very important to examine the *characteristics of the partners (spouses) of workaholics*, moreover, the possible relationship between *codependency* and work addiction should be investigated in future studies. 5) Though a very few amount of the existing studies already involved both the employees and his/her relatives or colleagues, it would be highly suggested to have more research applying *multi-rater techniques*. This way the biases caused by self-report measures would be better controlled. 6) Finally, much more longitudinal studies should analyze the associations between work addiction and the quality of social relationships. Although this research design is also insufficient to answer all the questions regarding causes and consequences, the temporal order of work addiction and social difficulties could be specified.

## Conclusion

The present systematic review and a series of meta-analyses clearly showed that work addiction is associated with an elevated level of problems in social relationships. Lower work-life balance, higher work-family conflicts, higher social dysfunctions, and lower quality of relationships and were found among people with work addiction. These findings emphasize the negative and maladaptive nature of work addiction contrary to the misconceptions that work addiction is a “positive addiction”. Findings of the current review reflect several core components of addictive disorders [17] showing that work addiction is very similar to other substance-related and behavioral addictions. The ‘Salience’, ‘Conflict’, ‘Problems’, and ‘Mood modification’ components can also be related to the problematic social relationships in work addiction. Since the quality of social relationships and social support are key factors in physical and mental health [74], the prevention and intervention of work addiction should be more accentuated in organizational and clinical settings.

## Supporting information

S1 Appendix PRISMA Checklist. (DOCX)

S2 Appendix Conceptual summary of the operational definition section. (DOCX)

S3 Appendix Data extraction form. (DOCX)

S4 Appendix Results of the moderator analyses regarding work addiction scales. (DOCX)

S1 Table Summary of studies included in the systematic review. (DOCX)

S2 Table Quality analysis of the primary articles included in the systematic review. (DOCX)

S3 Table Average effect sizes in the five meta-analysis and corresponding heterogeneity. (DOCX)

S1 Fig The number of specific work addiction measurement tools applied in the studies. (DOCX)

S2 Fig Funnel plot of the first meta-analysis: The relationship between work addiction and work-life imbalance. (DOCX)

S3 Fig Funnel plot of the second meta-analysis: The relationship between work addiction and difficulties in general social life. (DOCX)

S4 Fig Funnel plot of the third meta-analysis: The relationship between work addiction and difficulties in family life. (DOCX)

S5 Fig Funnel plot of the fourth meta-analysis: The relationship between work addiction and quality of intimate relationships. (DOCX)

S6 Fig Funnel plot of the fifth meta-analysis: The relationship between work addiction and quality of relationships with friends, community, and colleagues. (DOCX)
