# Supplementary material for: Work addiction and social functioning: A systematic review and five meta-analyses
Source: PLoS One. 2024 Jun 4;19(6):e0303563. doi: 10.1371/journal.pone.0303563 (PMC11149883; doi:10.1371/journal.pone.0303563)
Supplement: S2 Appendix — (DOCX) [file pone.0303563.s002.docx]

**S2 Appendix. Conceptual Summary of the Operational Definition Section.**

In the first category, *work-life balance (WLB)* refers to the arrangement of an individual's professional and personal life. This category includes the topics of “work-life conflict”, “work-life balance”, “work-life fit”, “work-nonwork conﬂict”, “work-nonwork enrichment”, “personal life interference with work”, and “work interference with personal life”. The second main category, *social functioning* includes general characteristics of social relationships, the quality of social life or the time spent with social activities. When examining the articles of this category, multiple global characteristics were distinguished, including “social dysfunction”, “quality of relationship”, “social health”, “time communicating with significant others”, “time spent on social activities”, “social optimism”, and “social pessimism”. The third main category refers to *family life*, including “family and leisure centrality”, “family interference with work”, “family functioning”, “family satisfaction”, “family engagement”, “family-to-work conflict”, “home-work conflict”, “negative work-family spillover”, “negative work–home/home–work interference”, “positive work-family spillover”, “work interference with family”, “work-family conflict”, “work-family facilitation”, “work-home conflict”, “work-to-family enrichment” and “family support”. The fourth category that also indicates family life but not in the same level, i.e., *family of origin and offspring of workaholics*, included the following topics: “health in the workaholic’s family of origin”, “perceived parental work addiction”, “work addiction of the offspring of workaholics”, the “mental health of the offspring of workaholics”, “time spent with children”, and “subjective experience of caretaking responsibility”. The fifth main category focuses on the *intimate* *relationship with the partner*, including “instrumental spousal support”, “quality of sex/intimacy”, “marital disillusionment”, “marital instability”, “marital disaffection”, “marital satisfaction” and “divorce”. Finally, the sixth category includes all the other important social relationships, namely *community, friends, and workplace relationships* including “social support”, “friend support”, “supervisory support”, “co-worker support”, “workplace support”, “relationship with the colleagues”, “conflicts with the colleagues”, “leader-member exchange”, “quality of friendships”, and “satisfaction with community life”.
