# Supplementary material for: Work addiction and social functioning: A systematic review and five meta-analyses
Source: PLoS One. 2024 Jun 4;19(6):e0303563. doi: 10.1371/journal.pone.0303563 (PMC11149883; doi:10.1371/journal.pone.0303563)
Supplement: S4 Appendix — (DOCX) [file pone.0303563.s004.docx]

**S4 Appendix. Results of the Moderator Analyses regarding Work Addiction Scales.**

**I. Family relationships**

The analysis was conducted for each of the work addiction scales, regarding the relationship between work addiction and difficulties in family life. As shown in Table A1, there is a significant positive relationship between work addiction and difficulties in family relationships in all cases. The strongest correlation, which is high, is observed with the BWAS scale, albeit based on only 3 studies. The WART and MWS (the latter based only on 3 studies) exhibit a medium-strong relationship, while the other scales demonstrate a weak positive relationship with family relationship difficulties.

**Table A1. Results of the moderator analyses focusing on the work addiction scales in context of family life.**

| **Scale** | **k** | **n** | **r** | **95% CI** | **Z** | **p** |
| --- | --- | --- | --- | --- | --- | --- |
| **DUWAS** | 26 | 20,837 | **.280** | 0.185, 0.369 | 30.254 | < .001 |
| **WART** | 11 | 11,978 | **.359** | 0.267, 0.445 | 7.197 | < .001 |
| **WorkBat** | 9 | 2982 | **.181** | 0.010, 0.343 | 2.071 | .038 |
| **BWAS** | 3 | 9328 | **.609** | 0.596, 0.621 | 68.249 | < .001 |
| **MWS** | 2 | 921 | **.383** | 0.287, 0.471 | 7.314 | < .001 |
| **WAQ** | 1 | 414 | **.272** | 0.180, 0.359 | 5.650 | < .001 |
| **WRI** | 1 | 587 | **.287** | 0.211, 0.359 | 7.129 | < .001 |

*Note*. BWAS, Bergen Work Addiction Scale; DUWAS, Dutch Work Addiction, Scale; MWS, Multidimensional Workaholism Scale; WART, Work Addiction Risk, Test; WAQ, Workaholism Analysis Questionnaire; WorkBat, Workaholism Battery; WRI, Work-Related Inventory. Significant correlations in bold.

**II. Work-life imbalance**

When the work addiction scales were analyzed separately, the results indicated a significant positive relationship with all three scales in the studies included (Table A2). While WorkBat and WART exhibit a medium correlation, DUWAS shows a weak positive correlation with the degree of work-life imbalance.

**Table A2. Results of the moderator analyses focusing on the work addiction scales in context of work-life imbalance.**

| **Scale** | **k** | **n** | **r** | **95% CI** | **Z** | **p** |
| --- | --- | --- | --- | --- | --- | --- |
| **WorkBat** | 6 | 1,318 | **.314** | 0.197, 0.423 | 5.071 | < .001 |
| **WART** | 2 | 544 | **.553** | 0.366, 0.697 | 5.109 | < .001 |
| **DUWAS** | 3 | 1,553 | **.206** | 0.155, 0.257 | 7.730 | < .001 |

*Note*. DUWAS, Dutch Work Addiction, Scale; WART, Work Addiction Risk, Test; WorkBat, Workaholism Battery. Significant correlations in bold.

**III. General social life**

In regard to the work addiction scales, significant correlations were observed in all cases within this meta-analysis. While the WAQ scale indicates a moderate relationship, the WART and WorkBat scales display weak but significant positive associations with difficulties in social life (Table A3).

**Table A3. Results of the moderator analyses focusing on the work addiction scales in context of general social life.**

| **Scale** | **k** | **n** | **r** | **95% CI** | **Z** | **p** |
| --- | --- | --- | --- | --- | --- | --- |
| **WAQ** | 4 | 875 | **.349** | 0.165, 0.510 | 3.611 | < .001 |
| **WART** | 4 | 863 | **.217** | 0.031, 0.388 | 2.280 | .023 |
| **WorkBat** | 2 | 937 | **.185** | 0.046, 0.317 | 2.590 | .010 |

*Note*. WART, Work Addiction Risk, Test; WAQ, Workaholism Analysis Questionnaire; WorkBat, Workaholism Battery. Significant correlations in bold.

**IV. Intimate relationships**

When analyzing the work addiction scales separately, only the WART and WAQ scales showed a significant negative relationship with intimate relationship quality, whereas the WorkBat and DUWAS scales did not (Table A4).

**Table A4. Results of the moderator analyses focusing on the work addiction scales in context of the quality of intimate relationships.**

| **Scale** | **k** | **n** | **r** | **95% CI** | **Z** | **p** |
| --- | --- | --- | --- | --- | --- | --- |
| **WART** | 7 | 1605 | **–.225** | –0.379, –0.059 | –2.643 | .008 |
| **WAQ** | 4 | 875 | **–.385** | –0.579, –0.150 | –3.120 | .002 |
| **WorkBat** | 3 | 498 | –.010 | –0.098, 0.078 | –0.222 | .824 |
| **DUWAS** | 1 | 244 | –.090 | –0.213, 0.036 | –1.401 | .161 |

*Note*. DUWAS, Dutch Work Addiction, Scale; WART, Work Addiction Risk, Test; WAQ, Workaholism Analysis Questionnaire; WorkBat, Workaholism Battery. Significant correlations in bold.

**V. Relationships with friends, community, and colleagues**

Regarding the specific work addiction scales, we found that only DUWAS and WAQ showed significant and negative correlation with the quality of relationship with friends, community, and colleagues (Table A5).

**Table A5. Results of the moderator analyses focusing on the work addiction scales in context of the quality of relationships with friends, community, and colleagues.**

| **Scale** | **k** | **n** | **r** | **95% CI** | **Z** | **p** |
| --- | --- | --- | --- | --- | --- | --- |
| **DUWAS** | 6 | 6,808 | **–.141** | –0.207, –0.072 | –4.013 | < .001 |
| **WorkBat** | 5 | 2165 | –.032 | –0.126, 0.062 | –0.670 | .503 |
| **WAQ** | 3 | 760 | **–.377** | –0.437, –0.314 | –10.872 | < .001 |
| **WART** | 1 | 587 | –.055 | –0.135, 0.026 | –1.331 | .183 |

*Note*. DUWAS, Dutch Work Addiction, Scale; WART, Work Addiction Risk, Test; WAQ, Workaholism Analysis Questionnaire; WorkBat, Workaholism Battery. Significant correlations in bold.
