# Supplementary material for: Work addiction and social functioning: A systematic review and five meta-analyses
Source: PLoS One. 2024 Jun 4;19(6):e0303563. doi: 10.1371/journal.pone.0303563 (PMC11149883; doi:10.1371/journal.pone.0303563)
Supplement: S1 Table — (DOCX) [file pone.0303563.s005.docx]

**S1 T****able. Summary of studies included in the systematic review.**

|  | **Study** | **Country** | **Research design** | **Sample characteristics** | | | **Work addiction measure (Cronbach’s α)** | **Social variable** | **Measure of social variable (Cronbach’s α)** | **Statistical analyses** | **Results of correlational analyses** | **Results of regression, mediation, or SEM analyses** | **Results of group comparison tests** |
| --- | --- | --- | --- | --- | --- | --- | --- | --- | --- | --- | --- | --- | --- |
|  |  |  |  | **Sample size** | **Mean age (SD)** | **% of males** |  |  |  |  |  |  |  |
| 1 | Andreassen et al 2013 | Norway | cross-sectional | 661 | 42.6 (10.5) | 46 | WorkBat  (Drive 0.83, Involvement 0.57; Enjoyment 0.85) | Negative W-F spillover | Work-Family Interface Scale (WFIS)  (Negative W-F spillover 0.93;  Positive W-F spillover 0.68;  Negative F-W spillover 0.87;  Positive F-W spillover 0.72) | Correlation, hierarchical multiple regression (outcome: WFIS) | Involvement: 0.23**; Drive: 0.32**;  Enjoyment: 0.09* | Involvement: β = 0.17**  Enjoyment: β = 0.03  Drive: β = 0.24** |  |
|  |  |  |  |  |  |  |  | Positive W-F spillover |  |  | Involvement: 0.03;  Drive: –0.10**; Enjoyment: 0.35** | Involvement: β = 0.04  Enjoyment: β = 0.37**  Drive: β = –0.10** |  |
|  |  |  |  |  |  |  |  | Negative F-W spillover |  |  | Involvement: 0.11**; Drive: 0.12**;  Enjoyment: 0.00 | Involvement: β = 0.05  Enjoyment: β = 0.03  Drive: β = 0.11** |  |
|  |  |  |  |  |  |  |  | Positive F-W spillover |  |  | Involvement: 0.10*;  Drive: 0.03;  Enjoyment: 0.20** | Involvement: β = 0.08*  Enjoyment (β = 0.19***  Drive: β = 0.01 |  |
| 2 | Ayar et al., 2022 | Turkey | cross-sectional | 336 | 31.56 (8.50) | 14 | WART (0.86) | Work-life balance | Work-Life Balance Scale (WLBS) (0.91) | Correlation, SEM (outcome: WLB) | –0.63* | direct effect: β = –0.84* |  |
| 3 | Aziz, Adkins, et al., 2010 | USA | cross-sectional | 208 | n/a | 41 | WART (0.89) | Work-life imbalance | Work-Life Imbalance Scale (WLIB) (0.89) | Correlation, multiple regression (outcome: work addiction) | 0.46** | β = 0.48** |  |
| 4 | Aziz & Cunningham, 2008 | USA | cross-sectional | 199 | n/a | 46 | WorkBat  (Drive 0.82; Involvement 0.72; Enjoyment 0.88) | Work-life imbalance | Work Interference with Personal Life Scale (WIPLS) (0.92) | Correlation, hierarchical multiple regression (outcome: workaholism) | Involvement: 0.23**; Drive: 0.36**;  Enjoyment: –0.13  Workaholism composite score: 0.44** | direct effect: β = 0.43**  Gender does not moderate the relation between WIPL and WorkBat |  |
| 5 | Aziz, Wuensch, et al., 2010 | USA | cross-sectional | 199 | n/a | 32 | WorkBat  (Drive 0.82; Involvement 0.72; Enjoyment 0.88) | Work-life imbalance | Work-Life Imbalance Scale (WLIB) (0.93) | Correlation, multiple regression (outcome: WLIB), REGWQ | Involvement: 0.18**;  Drive: 0.37**;  Enjoyment: –0.01; Workaholism composite score: 0.43** | β = 0.52** | workaholics had higher levels of WLIB (M=3.16) than work enthusiasts (M=2.50)*, unengaged workers (M=2.43)*, and relaxed workers (M=2.18 )* |
| 6 | Aziz & Zickar, 2006 | Canada & USA | cross-sectional | 174 | 43 (n/a) | 56 | WorkBat  (Drive 0.78-0.81; Enjoyment 0.88-0.90; Involvement 0.73-0.74) | Work-life imbalance | Work Interference with Personal Life Scale (WIPLS) (0.78) | Correlation | Among employees:  Involvement: 0.38**; Drive: 0.48**;  Enjoyment: –0.01  Among acquaintances:  Involvement: 0.43**; Drive: 0.42**;  Enjoyment: 0.18** |  |  |
| 7 | Babic et al., 2022 | Belgium | longitudinal, 3 waves | 464 | n/a | 40 | DUWAS  (0.84–0.89) | Work-family conflict | Survey Work-Home Interaction (SWING)  (0.85–0.86) | Correlation, SEM | DUWAS T1 with SWING  T1: 0.51**  T2: 0.44**  T3: 0.43**  DUWAS T2 with SWING:  T2: 0.50**  T3: 0.48**  DUWAS T3 with SWING  T3: 0.51** | Workload T1 → Work addiction T2 (β = 0.13**) → WFC T3 (β = 0.18**) |  |
| 8 | Bakker et al., 2009 | Netherlands | cross-sectional | 336 (168 couples) | men: 35.8 (4.59); women: 33.6 (3.88) | 50 | CT subscale of WART (0.80–0.81) | Work-family conflict | 3 item scale of Survey Work–Home Interference Nijmegen (SWING)  (0.74–0.77) | Correlation, SEM (outcome: WFC) | Men: 0.65**  Women: 0.67** | Men: β = –0.78**, women: β = –0.86**  WFC was mediator between men’s workaholism and  women’s experience of social support (*z*=–3.50**) and between women’s workaholism and men’s experience of social support (*z*=–3.60**) |  |
|  |  |  |  |  |  |  |  | Social support | Eight-item scale by Abbey et al. (0.87–0.88) |  | Men: –0.13  Women: –0.19* |  |  |
|  |  |  |  |  |  |  |  | Satisfaction in the current intimate relationship | Five-item scale of the Relational Interaction Satisfaction Scale (0.84–0.88) |  | Men: 0.04  Women: –0.09 |  |  |
| 9 | Bakker et al., 2013 | Netherlands | cross-sectional | 85 | 38.4 (9.3) | 56.5 | CT subscale of WART (0.82) | Time spent on social activities | DRM-diary | Correlation | –0.09 |  |  |
| 10 | Bakker et al., 2014 | Japan | longitudinal | 796  (398 couples) | Men: 38.1 (5.1) women: 36.3 (3.8) | 50 | DUWAS  (0.81–0.84) | Work-family conflict | Eight-item scale from Survey Work-Home Interference Nijmegen (SWING) (0.84) | Correlation, SEM (outcome: WFC) | Men: 0.59**  Women: 0.54** | men: β = –0.12*  women: β = –0.13*  Indirect effect of DUWAS on partner’s family satisfaction via WFC and own family satisfaction:  Men’s DUWAS on women’s family satisfaction: β = –0.02  Women’s DUWAS on men’s family satisfaction: β = –0.01 |  |
|  |  |  |  |  |  |  |  | Work-family facilitation | Five-item scale from Survey Work-Home Interference Nijmegen (SWING) (0.74–0.75) |  | Men: 0.06  Women: 0.13** |  |  |
|  |  |  |  |  |  |  |  | Family satisfaction | Single-item scale by Shimazu and Schaufeli (n/a) |  | DUWAS T1 with WFC T1 and T2:  Men T1: –0.11*; T2: –0.07  Women: T1: –0.10*;  T2: –0.12* |  |  |
| 11 | Balducci et al., 2017 | Italy | cross-sectional | 171 | n/a | 59.9 | DUWAS  (WE and WC: 0.74) | Work-family conflict | Two-item scale by Grzywacz et al. (n/a) | Correlation | DUWAS: 0.41**  WE: 0.44**  WC: 0.28** |  |  |
| 12 | Balkin et al., 2018 | USA | cross-sectional | 115 | 39.86 (12.36) | 40.9 | WAQ (0.98) | Quality of relationships | Juhnke–Balkin Life Balance Inventory (JBLI) (0.91–0.98) | Correlation, multiple regression (outcome: WAQ) | –0.63** | β = 0.10 |  |
|  |  |  |  |  |  |  |  | Sex/intimacy |  |  | –0.71** | β = –0.20 |  |
|  |  |  |  |  |  |  |  | Friendship |  |  | –0.87** | β = –0.35* |  |
|  |  |  |  |  |  |  |  | Community support |  |  | –0.89** | β = –1.07** |  |
| 13 | Bartczak & Ogińska-Bulik, 2012 | Poland | cross-sectional | 126 | 45.9 (11.3) | 48.4 | WART (0.87) | Social dysfunction | Social disfunction subscale of General Health Questionnaire GHQ-28 (0.93) | Correlation, hierarchical multiple regression (outcome: Social dysfunction) | Total WART: 0.22*  Subscales:  Obs/Comp.: 0.18**  Emot/Perf.: 0.19**  Overd.: 0.17  Res.: 0.34**  Self.: 0.05 | Obs/Comp.: β = 0.08  Emot/Perf.: β = 0.03  Overd.: β = 0.17  Res.: β = 0.33**  Self.: β = –0.16 |  |
| 14 | Bayhan Karapinar et al., 2020 | Turkey | cross-sectional | 244 | 36.51 (8.42) | 34 | DUWAS-10 (0.83) | Work-family conflict | Work-Family Conflict Scale (WFCS) (0.88) | Correlation, mediation analysis (outcome: WFC) | 0.34** | β = 0.51  WFC mediated between DUWAS-10 and well-being –0.08* |  |
|  |  |  |  |  |  |  |  | Instrumental spousal support | Fifteen item scale of Family Support Inventory (0.89) |  | –0.09 |  |  |
| 15 | Bonebright et al., 2000 | USA | cross-sectional | 175 | 39.5 (9.13) | 74 | WorkBat  (0.71–0.85) | Work-family conflict | Work Interference with Family Scale (WIFS) (0.80) | Correlation, t-test | Driven: 0.42**, Involvement: 0.20**  Enjoyment: –0.14 |  | Enthusiastic workaholics (M = 14.51) and Non-enthusiastic workaholics (M =14.57) have more work-life conflicts than non-workaholics (M = 10.84)* |
| 16 | Borges et al., 2021 | Portugal | cross-sectional | 729 | 38 (9.7) | 18 | DUWAS  (WE: 0.63;  WC: 0.75) | Negative work-family interaction | Survey Work-Home Interaction – Nijmegen (SWING) (0.796-0.896 | Correlation, multiple regression (outcome: DUWAS, WC, WE) | WC: 0.48**  WE: 0.62**  DUWAS: 0.62** | on WC: β = 0.49**  on WE: β = 0.62**  on DUWAS: β = 0.621** |  |
|  |  |  |  |  |  |  |  | Negative family-work interaction |  |  | WC: 0.15**  WE: 0.20**  DUWAS: 0.20** |  |  |
|  |  |  |  |  |  |  |  | Positive work-family interaction |  |  | WC: –0.20  WE: –0.09**  DUWAS: –0.06 | on WC: β = 0.08** |  |
|  |  |  |  |  |  |  |  | Positive family-work interaction |  |  | WC: 0.06  WE: 0.06  DUWAS: 0.07 | on WE: β = 0.08**  on DUWAS: β = 0.09** |  |
| 17 | Brady et al., 2008 | USA | cross-sectional | 129 | 47.3 (10.58) | 54 | WART (0.87)  WorkBat  (0.65-0.89) | Work-family conflict | Work-Family Conflict Scale (WFCS) (0.91) | Correlation, regression (outcome: WFC) | WART: 0.43**  Drive: 0.42**  Involvement: 0.16  Enjoyment: –0.12 | WART: β = 0.27*  Drive: β = 0.28*  Enjoyment: β = –0.24  Involvement: β = –0.07 |  |
|  |  |  |  |  |  |  |  | Relationship satisfaction | Relationship Assessment Scale (0.95) |  | WART: 0.04  Drive: –0.08  Involvement: –0.03  Enjoyment: 0.13 |  |  |
|  |  |  | cross-sectional | 103 | 41.4 (9.11) | 15 | WART (0.88)  WorkBat  (0.62-0.89) | Work-family conflict | Work-Family Conflict Scale (WFCS) (0.91) | Correlation, regression (outcome: WFC) | WART: 0.47**  Drive: 0.45**.  Involvement: 0.15  Enjoyment: –0.04 | WART: β = 0.37**  Drive: β = 0.27*  Enjoyment: β = –0.19*  Involvement: β = –0.02 |  |
|  |  |  |  |  |  |  |  | Relationship satisfaction | Relationship Assessment Scale (0.92) |  | WART: –0.21  Drive: –0.16  Involvement: –0.05  Enjoyment: –0.05 |  |  |
| 18 | Braun et al., 2019 | Brasil | cross-sectional | 275 | 40 (8.60) | 65.4 | DUWAS  (WC: 0.70; WE: 0.74) | Work interference with family (WIF) | Work-Family Conflict Scale (WFCS)  (WIF 0.90; FIW 0.85) | Correlation,  regression (outcome: WIF) | WE: 0.38**  WC: 0.32** | among women:  WE: β = 0.40**  among men:  WE: β = 0.29** |  |
|  |  |  |  |  |  |  |  | Family interference with work (FIW) |  |  | WE: 0.18**  WC: 0.13** |  |  |
| 19 | Burke, 1999 | Canada | cross-sectional | 530 | n/a | n/a | WorkBat  (Drive 0.80; Enjoyment 0.88; Involvement 0.67) | Family satisfaction | 7 item scale from Extra Work Satisfaction Scale (0.89) | Correlation, ANOVA | Drive: –0.10*  Involvement: –0.03  Enjoyment: 0.17** |  | Workaholics have less family satisfaction than do the other working types.  Workaholics have less friend satisfaction and community satisfaction than Work enthusiastic and Relaxed workers. |
|  |  |  |  |  |  |  |  | Friends satisfaction | 3 item scale from Extra Work Satisfaction Scale (0.85) |  | Drive: –0.13**  Involvement: –0.01  Enjoyment: 0.09* |  |  |
|  |  |  |  |  |  |  |  | Community satisfaction | 4 item scale from Extra Work Satisfaction Scale (0.80) |  | Drive: –0.21**  Involvement: 0.08  Enjoyment: 0.11* |  |  |
| 20 | Burke, 2000 | Canada | cross-sectional | 530 | n/a | n/a | WorkBat (n/a) | Divorce | Demographic questions: divorced and married managers were compared | Mann-Whitney U test, ANOVA |  |  | Divorced and married managers reported similar rates of workaholism (data n/a). The three workaholism profiles showed the same percentages of divorced individuals. |
| 21 | Burke et al., 2003 | Australia | cross-sectional | 658 | n/a | n/a | WorkBat (n/a) | Divorce | Demographic questions: divorced and married psychologist were compared | t-test |  |  | Divorced and married psychologists reported similar rates of workaholism (data n/a) |
| 22 | Burke & Fiksenbaum, 2009 | Canada | cross-sectional | 530 | n/a | 52.5 | 7 items of WorkBat (0.88) | Family satisfaction | 7 items from Extra Work Satisfaction Scale (0.89) | Correlation | –0.11* |  |  |
|  |  |  |  |  |  |  |  | Friends satisfaction | 3 items from Extra Work Satisfaction Scale (0.85) |  | –0.14** |  |  |
|  |  |  |  |  |  |  |  | Community satisfaction | 4 items from Extra Work Satisfaction Scale (0.80) |  | –0.24** |  |  |
| 23 | Caesens et al., 2014 | Belgium | cross-sectional | 343 | 28.28 (4.43) | 42.86 | DUWAS-10 (0.83) | Supervisor support | Survey of Perceived Organizational Support (SPOS) (0.90) | Correlation,  SEM (outcome: WA) | –0.17** | β = –0.05 |  |
|  |  |  |  |  |  |  |  | Coworker support | Survey of Perceived Organizational Support (SPOS) (0.89) |  | –0.19** | β = –0.15* |  |
| 24 | Chamberlin & Zhang, 2009 | USA | cross-sectional | 279 | 22.14 (4.81) | 30 | WART (0.89) | Perceived parental workaholism | Children of Workaholic Parents Screening Test (CWST) (0.90) | Correlation | 0.20** |  |  |
| 25 | Chang et al., 2022 | China | longitudinal | 322 | 32.42 (6.06) | 50.9 | DUWAS-10 (0.93) | Family-supportive supervisor behavior (FSSB) | FSSB-SF (Family Supportive Supervisor Behavior Short-Form (0.80) | Correlation, SEM (outcome: WFC) | WA Time 1 - FSSB Time 2:  –0.37** | interaction between WA and FSSB in predicting WFC (β = 0.19**) and psychological detachment from work (β = 0.36**) |  |
|  |  |  |  |  |  |  |  | Work-family conflict (WFC) | Work-Family Conflict Scale (0.92) |  | WA Time 1 - WFC Time 3:  0.38** | β = 0.39** |  |
| 26 | Clark et al., 2014 | USA | longitudinal | 340 | 46 (10.48) | 27 | CT subscale of WART (0.82) | Work-family conflict (WHC) | 4 items from Work–Home and Home–Work Conflict and Enrichment Scale  (WHC 0.74; HWC 0.64) | Correlation, multiple mediation analysis (outcomes: WHC and HWC) | 0.48** | direct path: β = 0.31**  mediated by: work guilt, work anxiety, work anger, work disappointment |  |
|  |  |  |  |  |  |  |  | Family-work conflict (HWC) |  |  | 0.37** | direct path: β = 0.25**  mediated by: work guilt, work anxiety, work anger, work disappointment |  |
|  |  |  |  |  |  |  |  | Work-family enrichment (WHE) | 3 items from Work–Home and Home–Work Conflict and Enrichment Scale  (WHE 0.73; HWE 0.71) |  | 0.11* |  |  |
|  |  |  |  |  |  |  |  | Family-work enrichment (HWE) |  |  | –0.09 |  |  |
| 27 | Converso et al., 2019 | Italy | cross-sectional | 291 | 47 (9.9) | 69.4 | DUWAS-10 (WE: 0.85; WC: 0.76) | Work-family conflict | Five-item scale from Survey Work – Home Interference Nijmegen (SWING) (0.89) | Correlation, multiple mediation analysis (outcome: WFC) | WE: 0.59**  WC: 0.47** | WE: β = 0.31*  WC: β = 0.17 * |  |
|  |  |  |  |  |  |  |  | Conflict with colleagues | Four items from the Multidimensional Organisational Health Questionnaire (0.88) |  | WE: 0.08  WC: 0.07 |  |  |
| 28 | Daniel et al., 2022 | France | cross-sectional | 307 | 41 (9.26) | 35.82 | DUWAS-10 (0.78) | Work-family conflict | Work-Family Conflict scale (WFCS) (0.89) | Correlation,  moderator analysis | 0.25** | The greater the level of mindfulness, the weaker the relationship between WA and WFC.  effect = − 0.14, t = − 2.39,  [− 0.27; − 0.02** |  |
|  |  |  |  | 715 | 42.7 | 39.6 | DUWAS-10 (0.78) | Work-family conflict | Work-Family Conflict scale (WFCS) (0.89) | SEM (outcome: WFC) |  | regular mindfulness practice moderates the relationship between WA and WFC; χ2 Δ(1) = 19.31**  mindfulness-based training moderates the positive relationship between WA and WFC; (2 Δ(1) = 15.01** |  |
| 29 | Del Líbano et al., 2012 | Spain | cross-sectional | 386 | n/a | 35 | DUWAS-10  (WE: 0.85; WC: 0.74) | Work-family conflict | Resources-Experiences-Demands scale (0.72) | Correlation, SEM (outcome: WFC) | WE: 0.61**  WC: 0.42** | DUWAS-10: β = 0.74** |  |
| 30 | Di Stefano & Gaudiino, 2018 | Italy | cross-sectional | 212 | 42.61 (11. 01) | 53.1 | DUWAS-10  (WE: 0.75; WC: 0.80) | Work interference with personal life (WIPL) | Work Interference with Personal Life scale (WIPLS) (0.92) | Correlation, SEM (outcomes: WIPL and PLIW) | WE: 0.48**  WC: 0.18** | DUWAS-10 as predictor  β = 0.56** |  |
|  |  |  |  |  |  |  |  | Personal life interference with work (PLIW) | Personal Life Interference with Work scale (0.73) | Correlation, SEM | WE: 0.19*  WC: 0.20 | DUWAS-10 as predictor  β = 0.26** |  |
| 31 | Dong et al., 2022 | China | longitudinal | 422 (211 employees + 211 leaders) | 27.6 (3.2) + 34.1 (3.9) | 44.4 + 51.7 | DUWAS-10 (0.96) | Work-family conflict | multidimensional measure of work–family conflict (0.94) | Correlation, SEM (outcome: employee’s WFC) | leader’s WA and employees WFC:  0.75** | leader’s WA predicts employee’s WFC  β = 0.24**  with the increase of leader’s WA, the predicted value of work connectivity behavior after-hours to WFC decreased |  |
| 32 | Eason et al., 2022 | USA | cross-sectional | 226 | 32 (9) | 28.8 | WART (0.91) | Work to family conflict (WFC) | Work-Family Conflict scale (WFCS) (0.84) | Correlation, χ^2^ statistics, multiple regression analysis | CT: 0.39  NC: 0.25**  WART: 0.44** | CT: β = 0.15  NC: β = 0.82*  WART: β = 0.44 | Significant difference in WFC among WART categories**:  low risk: M = 90.07 medium risk: M = 107.19  high risk: M = 133.91 |
|  |  |  |  |  |  |  |  | Family to work conflict (FWC) |  |  | CT: 0.02  NC: –0.02  WART: –0.01  WFC and WART: 0.30** |  |  |
| 33 | Falco et al., 2012 | Italy | cross-sectional | 486 (243 couples) | 54.7 (6.72) | 50 | DUWAS-10 self-rating (SR)  (WE: 0.76; WC: 0.81) | Work-family conflict | 2 items scale of Work Family Conflict, (authors n/a) (0.73) | Correlation | SR-WE: 0.32**  SR-WC: 0.13* |  |  |
|  |  |  |  |  |  |  | DUWAS-10 observer rating (OR)  (WE: 0.86; WC: 0.89) |  |  |  | OR-WE: 0.28**  OR-WC: 0.15** |  |  |
| 34 | Falco et al., 2022 | Italy | cross-sectional | 8,419 | n/a | 62.3 | BWAS (0.78)  DUWAS-10 (0.83) | Work-family conflict | Qu–Bo test (0.91) | Correlation, regression analysis (outcome: WFC) | BWAS: 0.61**  DUWAS: 0.48** | BWAS: β = 0.50**  DUWAS: β = 0.19** |  |
| 35 | Flowers et al., 2000 | USA | cross-sectional | 323 | 47.9 (10.4) | 0 | WART (n/a) | Marital disaffection | Marital Dissatisfaction Scale (0.97) | Correlation | 0.48** |  |  |
| 36 | Fujiwara et al., 2016 | Japan | longitudinal | 125 families (couples) | Males: 39.6 (6.3) Females: 38 (4.5) | 50 | DUWAS (n/a) | Time spent with children (n/a) | 1 item/question | SEM (outcome: time spent with children) |  | β = –0.14 |  |
| 37 | Gillet et al., 2017 | France | cross-sectional | 465 | 38.49 (13.07) | 39.14 | DUWAS-10  (WE: 0.75; WC: 0.76) | Work-family conflict | 3 items from Survey Work-Home Interference Nijmegen (SWING) (0.89) | Latent profile analysis, group comparisons |  |  | The level of WFC was significantly differ in the four profiles:  Very high WA > moderately high WA > moderately low WA > very low WA |
|  |  |  | cross-sectional | 780 | 37.03 (10.67) | 39.36 | DUWAS  (Working excessively 0.75; Working compulsively 0.76) | Work-family conflict | 3 items from Survey Work-Home Interference Nijmegen (SWING) (0.89) | Latent profile analysis, group comparisons |  |  | The level of WFC was significantly differ in the four profiles:  Very high WA > moderately high WA > moderately low WA > very low WA |
| 38 | Gillet et al., 2018 | France | cross-sectional | 160 | 31.26  (SD n/a) | 5 | DUWAS-10  (WE: 0.78; WC: 0.70) | Work-family conflict | 3 items (0.83) | SEM (outcome: WFC) |  | β = 0.71** |  |
|  |  |  | cross-sectional | 321 + 332 | S1: 36.5 (9.18);  S2: 35.92 (9.34) | S1: 17;S2: 8 | DUWAS  (S1: 0.88  S2: 0.84) | Work-family conflict | 3 items (S1: 0.85; S2: 0.89) | Latent profile analysis, group comparison |  |  | The level of WFC was significantly differ in the four profiles:  Workaholics > Engaged-workaholics > Engaged workers = Disengaged workers |
|  |  |  | cross-sectional | 283 | 34.13 (9.28) | 31 | DUWAS  (0.86) | Work-family conflict | 3 items scale (0.91) | Latent profile analysis,  regression analysis (outcome: WFC) |  | Workaholic profile:  β = 0.44**  Engaged profile:  β = 0.55**  Engaged-workaholic profile:  β = 0.58** |  |
| 39 | Gillet et al., 2021 | France | cross-sectional | 419 | 39.45 (11.66) | 12.65 | 4 items of DUWAS (0.62) | Work-family conflict (WFC) | 3 items (0.84) | Correlation SEM (outcome: WFC, FLS) | 0.65** | WA predicted WFC:  β = 0.53**  WFC mediated the relationship between WA and FLS |  |
|  |  |  |  |  |  |  |  | Family life satisfaction (FLS) | 1 item (n/a) |  | –0.12* |  |  |
| 40 | Gillet et al., 2022 | France | Cross-sectional  (3 studies) | S1: 343  S2: 654 + 247  S3: 153 + 359 | S1: 42.21 (10.88)  S2: 41.08 (8.28) +  43.28 (8.47)  S3:  42.61(10.46) + 38.04 (10.34) | S1: 37.9  S2: 91.4 + 28.7  S3: 9.2 + 24.5 | DUWAS-10  Study 1:  WC: 0.72; WE: 0.85  Study 2:  WC: 0.80; WE: 0.76  Study 3:  WC 0.75; WE: 0.77 | Supervisor support (SS) | Survey of Perceived Organizational Support (SPOS) (0.89)  (S1: 0.93 S2: 0.91) | ESEM, LPA, multinomial logistic regressions (outcome: SS) |  | WA: β = –0.27**  WC: β = –0.14  WE: β = –0.20 | Study 2:  4 profiles:  P1: Low Global and Specific WA  P2: Average Global and Specific WA;  P3 Low Global and Average Specific WA);  P4: High Global and Average Specific WA.  LMX:  P1 > P4**  P3 > P4*  P1 > P2**  Study 3:  4 profiles:  P1: Average Global and Specific WA ;  P2: High Global and Average Specific WA;  P3: Low Global and Average Specific WA;  P4 (Nurses): Low Global WA/High Specific WA and Psychological Detachment;  P4 (Educators): Average Global WA/Low Specific WA and Psychological Detachment.  Supervisor support in S1:  P1 < P3**  P1 < P2**  P2 < P4**  P3 < P4**  S3:  WFC: P2 > P1 > P3** |
|  |  |  |  |  |  |  |  | Leader-member exchange (LMX) | Multidimensional LMX Scale |  |  |  |  |
|  |  |  |  |  |  |  |  | Work-family conflict | Work-Family Conflict Scale  (0.87) |  |  |  |  |
| 41 | Grawitch et al., 2018 | USA | cross-sectional | 742 | n/a | 47 | DUWAS-10 (0.83) | Work-life balance (WLB) | Satisfaction with Work-Life Balance Scale (0.94) | Correlation, multiple regression (outcome: WLB) | –0.17* | β = –0.09** |  |
| 42 | Hakanen & Peeters, 2015 | Finland | longitudinal | 1,580 | 44.41 (n/a) | 24.4 | CT subscale of WART  (T1: 0.80;  T2: 0.81;  T1: 0.80) | Work-to-family enrichment | 3 items from Work–Home and Home–Work Conflict and Enrichment Scale (T1: 0.74; T2: 0.78; T1: 0.81) | Correlation, SEM (outcome: WA and WFC) | Work addiction T1  T1: –0.02  T2: –0.04  T3: –0.00  Work addiction T2:  T2: –0.07  T3: –0.04  Work addiction T3:  T3: –0.08 | Work addiction T1 predicted WFC T2: β = 0.09**  Work addiction T2 predicted WFC T3: β = 0.22**  WFC did not predict future work addiction, neither from T1 to T2 nor from T2 to T3 |  |
|  |  |  |  |  |  |  |  | Work-family conflict | 4 items from Work–Home and Home–Work Conflict and Enrichment Scale (T1: 0.85; T2: 0.86; T3: 0.87) |  | Work addiction T1  T1: 0.53**  T2: 0.40**  T3: 0.40**  Work addiction T2:  T2: 0.54**  T3: 0.45**  Work addiction T3:  T3: 0.58** |  |  |
| 43 | Hamilton Skurak et al., 2021 | New-Zealand | cross-sectional | 162 | 41.20 (12.01) | 31.5 | Drive subscale of the WorkBat  (0.86) | Work-life conflict | Work-life conflict scale (0.89) | Correlation, SEM (outcome: WLC) | 0.54*** | β = 0.17**  WLC mediated between Drive and wellbeing  β = −0.06** |  |
| 44 | Hancock et al., 2019 | USA | cross-sectional | 245 | 41.2 (11.1) | 45.3 | WAQ (0.92) | Friendship | Juhnke–Balkin Life Balance Inventory (Friendship 0.75;  Quality of relationships 0.90;  Sex, intimacy 0.86) | Correlation, multiple regression analysis (outcome: WAQ) | –0.39*** | β = –0.16* |  |
|  |  |  |  |  |  |  |  | Quality of relationships |  |  | –0.28*** | β = –0.15* |  |
|  |  |  |  |  |  |  |  | Sex, intimacy |  |  | –0.22*** | β = 0.10 |  |
| 45 | Hauk & Chodkiewicz, 2013 | Poland | longitudinal | 178 | 43.4 (8.42) | 39.89 | Excessive Work Involvement Scale (SZAP) (0.60);  Scale of Workaholism as Behavioral Tendencies (SWBT) (0.87) | Work-family conflict (WFC) and family-work conflict (FWC) | Work-Family Conflict Scale (WFCS 0.94; FWC 0.80) | Regression (outcome: WFC and FWC), mediation analysis |  | SZAP predicts WFC: β = 0.46**  SZAP predicts FWC: β = 0.23**  SWBT predicts WFC: β = 0.14*  SWBT predicts FWC: β = 0.167*  General stress weakens the relationship between SZAP and WFC, FWC. |  |
| 46 | Hirschi et al., 2019 | Germany | longitudinal | 599 | 53.9 (2.7) | 55.6 | DUWAS-10  (T1: 0.88;  T2: 0.89) | Work–nonwork enrichment (WNE) | Work/Nonwork Scale, work–enhancement of personal life subscale  (T1: 0.82;  T2 :0.84) | Correlation, regression | Work addiction T1:  T1: 0.11**  T2: 0.08  Work addiction T2:  T1: 0.04  T2: 0.17** | Changes in WA predict changes in WNE: β = 0.22**, and WNC: β = 0.30** |  |
|  |  |  |  |  |  |  |  | Work–nonwork conflict (WNC) | Work/Nonwork Scale, work–interference with personal life subscale  (T1: 0.93;  T2: 0.94) |  | Work addiction T1:  T1: 0.53**  T2: 0.44**  Work addiction T2:  T1: 0.40**  T2: 0.49** |  |  |
| 47 | Hogan et al., 2016 | Ireland | cross-sectional | 410 | n/a | 50.2 | WorkBat-R  (Drive 0.75; Enjoyment 0.83) | Work-life conflict (WLC) | 4 items from Work-Life Conflict Scale (n/a) | Correlation, ANOVA | Drive: 0.42**  Enjoyment: –0.17** |  | Workaholics have higher WLC (M = 7.3) than relaxed workers (M = 5.9) and uninvolved workers (M = 6.5)** |
|  |  |  |  |  |  |  |  | Work-life fit (WLF) | Fit Scale (0.78) |  | Drive: –0.28**  Enjoyment: 0.32** |  | Workaholics have lower WLF (M = 16.1) than enthusiastic workaholics (M = 18.5) and relaxed workers (M = 21.8) |
| 48 | Huml et al., 2021 | USA | cross-sectional | 4,167 | 35.8 (11.11) | 54.9 | WART (0.81) | Work-family conflict | Work- Family Conflict Scale (WFCS) (0.92) | Correlation, SEM (outcome: WART) | 0.51* | β = 0.69**  Work-family conflict mediated between work engagement and work addiction: β = –0.17** |  |
| 49 | Huyghebaert-Zouaghi et al., 2022 | France | cross-sectional | 241 | 40.98 (11.07) | 3.31 | DUWAS-10  (WE: 0.79;  WC: 0.79) | Work-family conflict | Work-Family Conflict Scale (0.86) | ESEM and bifactor-ESEM (outcome: WFC) |  | DUWAS-10: β = 0.55**  WE: β = 0.05  WC: β = 0.34** |  |
|  |  |  | cross-sectional | 304 | 37.68 (12.06) | 29.6 | DUWAS-10  (WE: 0.76;  WC: 0.80) | Work-family conflict | Work-Family Conflict Scale (0.88) | ESEM and bifactor-ESEM (outcome: WFC) |  | DUWAS-10: β = 0.63**  WE: β = 0.34  WC: β = 0.37 |  |
| 50 | Innanen et al., 2014 | Finland | longitudinal | 161 | n/a | 26.4 | WART  (T2: 0.80;  T3: 0.84) | Social pessimism (SP) | Strategy and Attribution Questionnaire (SAQ) (SO - T1: 0.83; SP - T1: 0.85) | LPA, binary logistic regression (outcome: workaholic profile) |  | SP predicted Exhausted -workaholic profile  β = 1.52* |  |
| 51 | Kasemy et al., 2020 | Egypt | cross-sectional | 1,080 | 35.60 (10.34) | n/a | DUWAS (0.83) | Social health | World Health Organization Quality-of-Life Scale (WHOQOL-BREF) (0.70-0.80) | Multiple regression (outcome: social dysfunction, social healthy) |  | WC: β = –0.23*  WE: β = –0.41* |  |
|  |  |  |  |  |  |  |  | Social dysfunction | General Health Questionnaire (GHQ-28) (n/a) |  |  | WC: β = 0.19* |  |
| 52 | Kim et al., 2021 | South-Korea | cross-sectional | 496 | 41.14 (9.02) | 49 | Multidimensional Workaholism Scale (MWS)  (Motiv.: 0.83,  Cogn.: 0.91; Emot.: 0.84; Behav.: 0.90) | Work-family conflict | Work- Family Conflict Scale (WFCS) (0.93) | Correlation,  Multiple regression (outcome: WFC) | MWS with WFC:  Total score: 0.51**  Motivational: 0.36**  Cognitive: 0.47**  Emotional: 0.29**  Behavioral: 0.49** | Motiv.: β = 0.12**  Cogn.: β = 0.24**  Emot.: β = –0.07  Behav.: β = 0.31** |  |
| 53 | Kochanska et al., 2004 | USA | longitudinal | 112 mothers and 56 daughters | Mothers: 31 (n/a) daughters: 9 months (n/a) | 0 | Workaholism scale of SNAP (n/a) | Shared positive affective ambience (SPAA) | The parent’s and infant’s emotions were coded for each 30-s segment of interactions (0.78) | Hierarchical multiple regression (outcome: SPAA) |  | β = 0.24* |  |
| 54 | Kravina et al., 2010 | Italy | cross-sectional | 710 | n/a | 77.7 | DUWAS-10 (n/a) | Work-life conflict | Organizational Conflict Scale (n/a) | ANOVA |  |  | Workaholics have more work-life conflict (M = 4.20) than non-workaholics (M = 2.28), hard workers (M = 3.28), and compulsive workers (M = 2.50)** |
|  |  |  | cross-sectional | 710 | n/a | 77.7 | DUWAS-10 (n/a) | Conflict with superiors |  |  |  |  | Workaholics have more conflicts with superiors (M = 4.28) than non-workaholics (M = 2.09) and compulsive workers (M = 1.93)** |
| 55 | Kravina et al., 2014 | Italy | cross-sectional | 438 (146 families) | 44.6 (n/a)  parents: 53.4 (n/a)  adult children: 26.9 (n/a) | 47.5  parents: 50  children: 57.5 | DUWAS-10 score of the parents  (WC: 0.82;  WE: 0.74) | work addiction of the offspring | DUWAS-10 score of the adult children  (WC: 0.82;  WE: 0.74) | Multiple linear regression logistic regression  (outcome: child’s WE and WC) |  | Hierarchical regression for the child’s WE  Father’s WE: β = 0.36**  Father’s WC: β = –0.11  Mother’s WE: β = 0.14  Mother’s WC: β = 0.11  Hierarchical regression for the child’s WC  Father’s WE: β = 0.10  Father’s WC: β = –0.45  Mother’s WE: β = –0.41  Mother’s WC: β = 0.19  Logistic regression for the child’s work addiction  Father’s WE: OR = 2.71**  Father’s WC: OR = 0.82  Mother’s WE: OR = 1.51  Mother’s WC: OR = 1.39 |  |
| 56 | Lanaj et al., 2021 | USA | cross-sectional | 80 | n/a | 33.7 | CT subscale of WART (0.86) | Conflict at home in the evening | Three items for conflicts at home (0.81) | Correlation, | 0.10 |  |  |
| 57 | Levy, 2015a | USA | cross-sectional | 350 | 42.6 (7.8) | 0 | Drive and Enjoyment scales of WorkBat  (Drive: 0.77; Enjoy: 0.88) | Social support | Multi-dimensional Scale of Perceived Social Support (MSPSS) (0.91) | Correlation | Drive: −0.06  Enjoyment: 0.16** |  |  |
| 58 | Levy, 2015b | USA | cross-sectional | 266 | 43.8 (8.3) | 0 | WorkBat  (Drive 0.76;  Enjoyment 0.88; Involvement 0.60) | Marital satisfaction | Kansas Marital Satisfaction Scale (KMSS) (0.97) | Correlation,  hierarchical regression (outcome: KMSS) | Drive: −0.08  Enjoyment: 0.17**  Involvement: −0.10 | Enjoyment: β = 0.22**  Involvement: β = −0.14*  Drive: β =−0.06 |  |
| 59 | Loscalzo, 2021 | Italy | cross-sectional | 587 | 48.80 (9.17) | 5.5 | Workaholism scale of the Work-related Inventory (WI-10) (n/a) | Work-family conflict | Work-Family Conflict Scale (WFCS) (n/a) | Correlation, path analysis (outcome: WFC) | WFC Time: 0.29**  WFC Strain: 0.27**  WFC Behavior: 0.30** | WFC Time: β = 0.19**  WFC Strain: β = 0.23**  WFC Behavior: β = 0.27** |  |
| 60 | Lundkvist et al., 2016 | Sweden Scotland England Australia | cross-sectional | 261 | 42.8 (n/a) | n/a | DUWAS-10 (0.85) | Negative work–home interference (NWHI) | Short version of Survey Work–Home Interference Nijmegen (SWING) (NWHI 0.85; NHWI 0.70) | Correlation | 0.60** |  |  |
|  |  |  |  |  |  |  |  | Negative home–work interference (NHWI) |  |  | 0.25** |  |  |
| 61 | Mazzetti et al., 2019 | Italy | cross-sectional | 1,065 | 45.36 (8.23) | 57.4 | DUWAS-10 (0.81) | Work–family conflict | 3 item scale from Work-Family Conflict Scale (WFCS (0.91) | Correlation, moderated mediation (outcome: WFC) | 0.43** | β = 0.89**  Indirect effect of DUWAS-10 on WFC through presenteeism at low  (β = 0.03**), medium  (β = 0.02**), and high (β = 0.00) levels of managerial support. |  |
|  |  |  |  |  |  |  |  | Managerial support | 5 item scale from HSE Indicator Tool (0.84) |  | −0.12** |  |  |
| 62 | Mcmillan et al., 2004 | New Zealand | longitudinal | T1: 88;  T2: 42  Partners:  T1: 40  T2: 24 | Workaholics (Wcs): 37.5 (11.8); Non-workaholics (Nwcs): 30.5 (9.0)  and their partners:  38.1 and 33.1 | Workaholics: 50  Non-workaholics: 55 | WorkBat-R  (Drive 0.75;  Enjoyment 0.90) | Time communicating with significant others – self-ratings (SR) and ratings of significant others (OR) | Four-component items by authors (n/a) | Mann-Whitney U test |  |  | T1:  Wcs rated higher (M = 19.3) than their partners (M = 18). (ns)  Nwcs rated lower (M = 19.6) than their partners (M = 23.1). (ns)  T2:  Wcs rated lower (M = 15) than their partners (21.2). (ns)  Nwcs rated lower (M=15.3) than their partners (24.8). (ns) |
|  |  |  |  |  |  |  |  | Dyadic adjustment | Dyadic Adjustment Scale (DAS) (0.89) |  |  |  | T1:  Wcs reported higher (M =98.2) thank Nwcs (M = 96.8) (ns)  Wcs’ partners reported lower (M =97.4) than Nwcs’ partners (M =99.0) (ns)  Wcs dyads showed same level of adjustments (M = 97.7) thank Nwcs dyads (M = 99.2) (ns)  T2:  Wcs rated similar adjustment (M = 100.6) as Nwcs (M = 100.2) (ns)  Partners of Wcs rated similar (M = 99.2) as Nwcs’ partners (M = 100.3) (ns) |
| 63 | McMillan & O’Driscoll, 2004 | New Zealand | longitudinal | T1: 88;  T2: 56 | Workaholics Wcs): 37.5 (11.8); Non-workaholics (Nwcs): 30.5; (9.0) | Wcs: 50, Nwcs: 55 | WorkBat-R  (Drive 0.75; Enjoyment 0.90) | Social health (social functioning) | 14 items from Rand Short Form-36 (SF-36) (n/a) | Mann-Whitney U test |  |  | T1  Wcs reported lower level (M = 83.2) than Nwcs (M = 87.8) (ns)  T2  Wcs reported lower level (M = 78.6) than Nwcs (M = 88.4) (ns) |
| 64 | Molino et al., 2016 | Italy | cross-sectional | 617 | 39.95 (9.56) | 45 | BWAS (0.78) | Work-family conflict | Work-Family Conflict Scale (WFCS) (0.91) | Correlation, SEM | 0.59** | job demands 🡪 DUWAS 🡪 WFC: β = 0.29**  JD 🡪 DUWAS 🡪 WFC 🡪 Exhaustion: β = 0.06**  DUWAS 🡪 WFC 🡪 Exhaustion 🡪 Intentions to change job: β = 0.04** |  |
| 65 | Molino et al., 2022 | Italy | longitudinal | 292 | 43.62 (11.78) | 47.6 | BWAS (0.83)  DUWAS (0.79) | Work-family conflict | Work-Family Conflict Scale (WFCS) (0.90) | Correlation | DUWAS T1 with WFC T2:  0.23**  BWAS T1 with WFC T2:  0.56**  BWAS T2 with WFC T2:  0.66** |  |  |
| 66 | Morkevičiūtė & Endriulaitienė, 2022 | Lithuania | cross-sectional | 537 | 28.05 (6.84) | 30 | BWAS (0.84) | perceived work addiction of father/mother | BWAS (father: 0.93; mother: 0.92) | Correlation; SEM (outcome: work addiction of the adult child) | father: 0.25**  mother: 0.27** | father: β = 0.12**  mother: β = 0.22**  Extrinsic motivations mediated between father's WA and the child’s WA.  Extrinsic motivations of the mother and intrinsic motivations ow both parents did not mediate between the parent’s and offspring’s WA. |  |
| 67 | Niehuis, 2007 | USA | cross-sectional | 116 | Females: 35 (14.4); Males: 41 (14.5) | 36 | WART (0.89) | marital disillusionment | Marital Disillusionment Scale (0.96) | Correlation | 0.18 |  |  |
| 68 | Omar et al., 2021 | Argentina | cross-sectional | 459 | 37 (4.86) | 52.3 | DUWAS-10  (WE: 0.70;  WC: 0.68) | Work-family enrichment (WFE) | Work-Family Enrichment Scale (WFES: 0.81; FWES: 0.78) | Correlation | WE: −0.41**  WC: −0.44** |  |  |
|  |  |  |  |  |  |  |  | Family work enrichment (FWE) |  |  | WE: −0.37**  WC: −0.40** |  |  |
| 69 | Peplińska et al., 2015 | Poland | cross-sectional | 260 | n/a | 0 | WART (0.87) | Social support | Berlin Social Support Scale (BSSS) (Perceived support 0.90, The need for support 0.74, Seeking support 0.77, Support actively received 0.85, Protective support 0.80). | Correlation, Mediation analysis (outcome: work addiction) | In total sample:  −0.45**  Among women in a relationship with children: –0.18  Among single women with children: –0.83**  Among childless women in a relationship: –0.40*  Among single childless women: –0.52** | β = – 0.45**  Purpose in life mediated between social support and work addiction. |  |
| 70 | Quiñones-García & Korak-Kakabadse, 2014 | UK | cross-sectional | 516 | 41.7 (14.5) | 50.2 | DUWAS  (WE: 0.73;  WC: 0.79) | Social support | Five-items from Social Support Scale (0.86) | SEM |  | Interaction of social support on the relationship between WE and compulsive internet use  β = 0.09** |  |
| 71 | Rai et al., 2022 | India | longitudinal | 282 + 250 | 32.91 (5.83);  32.72 (4.74) | 64.9;  57.6 | DUWAS  (WE: 0.92; WC: 0.89) | Work-family conflict | Work-Family Conflict Scale (0.92) | Correlation, SEM (outcome: WFC) | Sample 1:  WE T1 - WFC T2: 0.32**  WC T1 - WFC T2: 0.17**  Sample 2:  WE T1 - WFC T2: 0.52**  WC T1 - WFC T2: 0.35** | Competitive climate positively relates to WFC through serial mediating effects of WE and WC and emotional exhaustion. |  |
| 72 | Reiner et al., 2019 | USA | cross-sectional | 409 | 45.9 (10.62) | 19.1 | WAQ (0.92) | Quality of relationships, | Life Balance Inventory (JBLI) (Quality of relationships 0.91; Sex/intimacy 0.73; Friendship 0.76) | Correlation, multiple regression analysis (outcome: WAQ) | –0.28** | β = –0.09 |  |
|  |  |  |  |  |  |  |  | Sex/intimacy |  |  | –0.30** | β = –0.08 |  |
|  |  |  |  |  |  |  |  | Friendship |  |  | –0.35** | β = 0.02 |  |
| 73 | Robinson & Carroll, 1999 | USA | cross-sectional | 207 | 25.1 (n/a) | 12.1 | Children of Workaholics Screening Test (CWST) (n/a) | Parentification of the child | Parentification Questionnaire (n/a) | Correlation | 0.60** |  |  |
| 74 | Robinson, Carroll, et al., 2001 | USA | cross-sectional | 326 | 47.9 (10.44) | 0 | WART (n/a) – observer rating, partner’s WA rated by the spouse | Marital disaffection | Marital Disaffection Scale (MDS) (0.97) | T-test |  |  | Spouses of workaholics showed higher marital estrange (M = 49.2) thank spouses of non-workaholics (M = 33.3) * |
|  |  |  |  |  |  |  |  | Overall affect toward spouse | Positive Feelings Questionnaire (PFQ) (n/a) |  |  |  | Spouses of workaholics showed lower positive feelings (M = 74.8) thank spouses of non-workaholics (M = 99.8)* |
| 75 | Robinson, Flowers, et al., 2001 | USA | cross-sectional | 326 | 47.9 (10.44) | 0 | WART  (CT: 0.89;  Control: 0.86;  IC: 0.68; | Marital disaffection | Marital Disaffection Scale (MDS) (n/a) | Correlation,  SEM (outcome: marital cohesion) | CT: 0.19**  Control: 0.50**  IC: 0.64**  SW: 0.25** | β = –0.77**  (Marital cohesion included marital affection and positive feelings toward the spouse.) |  |
|  |  |  |  |  |  |  | SW: 0.40);  observer rating, partner’s WA rated by the spouse | Overall affect toward spouse | Positive Feelings Questionnaire (PFQ)  (Positive feeling: 0.94;  Physical attraction: 0.96) |  | Positive feeling:  CT: –0.20**  Control: –0.55**  IC: –0.66**  SW: –0.29**  Physical attraction:  CT: –0.16**  Control: –0.47**  IC: –0.61**  SW: –0.24** |  |  |
| 76 | Robinson & Kelley, 1998 | USA | cross-sectional | 211 | 24 (n/a) | 22 | WART (n/a) | Depression at children of workaholic parents (CoWP) and children of non-workaholic parents (CoNWP) | Depression Inventory (BDI) (n/a) | T-test |  |  | CoWP (M = 11.0) showed higher depression than CoNWP (M = 8)*  CoW fathers (M = 13.16) showed higher depression than CoNW fathers (M = 7.81)*  No significant difference between CoW mothers (M = 8.85) and CoNW mothers (M = 8.52) |
|  |  |  |  |  |  |  |  | Self-concept of and CoNWP | Personal Attribute Inventory (n/a) |  |  |  | No significant difference between CoWP (M = 5.23) and CoNWP (M = 4.56)  No significant difference between CoW mothers (M = 5.45) and CoNW mothers (M = 4.61) |
|  |  |  |  |  |  |  |  | Locus of control of CoWP and CoNWP | Nowicki-Strickland Scale (NRS) (n/a) |  |  |  | CoWP (M = 9.41) showed higher external locus of control than CoNWP (M = 8.04)*  CoW fathers (M = 10.66) showed higher external locus of control than CoNW fathers (M = 8.06)*  No significant difference between CoW mothers (M = 8.82) and CoNW mothers (M = 8.35) |
|  |  |  |  |  |  |  |  | Anxiety of CoWP and CoNWP | State Trait Anxiety Scale X-2 (STAI) (n/a) |  |  |  | No significant difference between CoWP (M = 44.21) and CoNWP (M = 41.67)  CoW fathers (M = 46.56) showed higher anxiety than CoNW fathers (M = 41.70)*  No significant difference between CoW mothers (M = 43.31) and CoNW mothers (M = 42.21) |
| 77 | Robinson & Kelley, 1999 | USA | cross-sectional | 40 (children and their parents) | Children: 10.6 (n/a) | 40 | WART (among parents) (n/a) | Self-esteem  Locus of Control  Anxiety  Work addiction (among children) | The Coopersmith Self-Esteem Inventory (n/a)  Nowicki-Strickland Scale (n/a)  State-Trait Anxiety Inventory for Children (n/a)  Children's Version of the Work Addiction Risk Test (n/a) | Correlation | No significant correlation between parents’ work addiction and any of the child’s self-rated scores, i.e., self-esteem, locus of control, anxiety, and work addiction. |  |  |
| 78 | Robinson & Post, 1995 | USA & Canada | cross-sectional | 107 | 44 (n/a) | 40 | WART (n/a) | Self-perceived level of health in one's family of origin | Family of origin scale (FOS) (n/a) | Correlation, χ^2t^ test | FOS-Intimacy: 0.01  Autonomy: 0.06 |  | The social functioning of men was affected by work greater than among women (F = 9.87, p<.001) |
|  |  |  |  |  |  |  |  | Family functioning | McMaster Family Assessment Device (FAD) (n/a) |  | Problem solving: 0.30**  Communication: 0.34**  Roles: 0.36**  Affective responsiveness: 0.28*  Affective involvement: 0.25*  Behavioral control: 0.03  General functioning: 0.32** |  |  |
| 79 | Robinson & Post, 1997 | USA | cross-sectional | 107 | 44 (n/a) | 40 | WART (n/a) | Family functioning | McMaster Family Assessment Device (FAD) (n/a) | ANOVA |  |  | High risk group showed more problems than lower risk group on:  - problem-solving issues (M = 2.6 vs. M = 2.2 and M = 2.3)*  - communication (M = 2.8 vs. M =2.3 and M = 2.4)*  - affective responsiveness (M = 2.8 vs. M = 2.2 and M = 2.3)  - affective involvement (M = 2.7 vs. M = 2.3 and M = 2.3)*  - general family functioning (M = 2.7 vs. M = 2.1 and M = 2.2)* |
| 80 | Robinson et al., 2006 | USA | cross-sectional | 272 | 49.3 (9.5) | 100 | WART  (CT: 0.84;  Control: 0.85;  IC: 0.62;  Deleg.: n/a  SW: 0.53);  observer rating, partner’s WA rated by the spouse | Marital disaffection | Marital Disaffection Scale (MDS) (n/a) | Correlation, hierarchical multiple regression | WART Total: 0.36**  CT: 0.19**  Control: 0.36**  IC: 0.38**  Deleg.: 0.16**  SW: 0.15* | CT: β = –09  Control: β = 0.25**  IC: β = 0.27**  Deleg.: β = 0.09  SW: β = –0.06 |  |
| 81 | Ruiz-Garcia et al., 2022 | Spain | cross-sectional | 219 | 40.9 (10.6) | 9.1 | DUWAS-10 (n/a) | Negative work-family Interaction | Survey Work- Home Interaction Nijmegen (SWING) (n/a) | U Mann-Whitney Test |  |  | Workaholics (M = 2.44) showed higher conflict than nonworkaholics (M = 1.89).** |
|  |  |  |  |  |  |  |  | Negative family-work Interaction |  |  |  |  | Workaholics (M = 1.33) showed higher conflict than nonworkaholics (M = 1.28).** |
|  |  |  |  |  |  |  |  | Positive work-family Interaction |  |  |  |  | Workaholics (M = 2.48) showed higher interaction than nonworkaholics (M = 2.24).* |
|  |  |  |  |  |  |  |  | Positive family-work Interaction |  |  |  |  | Workaholics (M = 2.95) showed higher interaction than nonworkaholics (M = 2.67).* |
| 82 | Russo & Waters, 2006 | Australia | cross-sectional | 169 | 40.52 (8.18) | 33.7 | WorkBat  (Drive 0.85; Enjoyment 0.88; Involvement 0.65) | Work-family conflict | Work-family Conflict Scale (0.73) | Correlation, ANCOVA | Drive: 0.46**  Enjoyment: –0.09 |  | Workaholics experienced higher levels of WFC (M = 3.27) than relaxed workers (M = 2.85)**, but showed the same level as uninvolved workers (M = 3.03) and enthusiastic workaholics (M = 3.35) |
|  |  |  |  |  |  |  |  | Supervisor support | Supervisor Support Scale (0.74) |  | Drive: 0.01  Enjoyment: 0.18* |  | No difference in the level of WFC between the four worker types with the increasing levels of supervisor support. |
| 83 | Sawhney et al., 2022 | USA | cross-sectional,  across 10 days | 169 | 38.02 (9.97) | 40.24 | DUWAS-10 (between 0.86-0.92 across the 10 days) | daily work-family conflict (WFC) | Work-Family Conflict Scale (0.92-0.94 across the 10 days) | Correlation, regression, interaction  (outcomes: WFC and FE) | trait WA:  0.16**  daily WA:  0.17** | trait WA: β = 0.19**  daily WA: β = 0.10* |  |
|  |  |  |  |  |  |  |  | daily family engagement (FE) | Work and Family Engagement Survey (WFES) (0.94-0.96 across the 10 days) |  | trait WA:  0.19**  daily WA:  0.16** | trait WA: β = 0.15**  daily WA: β = 0.00 |  |
| 84 | Scafuri Kovalchuk et al., 2019 | Italy | cross-sectional | 395 | 39.98 (12.11) | 38 | DUWAS-10 (0.83) | Work-family conflict | Work-Family Conflict Scale (WFCS) (0.88) | Correlation, conditional process analysis (outcome: WFC) | 0.40 ** | β = 0.36**  Interaction between DUWAS and work engagement on WFC  (β = −0.20)**  If work engagement is low and DUWAS is high, WFC is higher than when work engagement is high. |  |
| 85 | Schaufeli et al., 2008 | Netherlands | cross-sectional | 587 | n/a | 78 | WE subscale of the WART (0.77)  Drive subscale of the WorkBat  (0.85) | Negative reactions | self-constructed items (0.72) | Correlation,  Multiple regression analysis (outcome: work addiction) | WE: 0.05  Drive: 0.24** | WE: β = 0.07  Drive: β = 0.18** |  |
|  |  |  |  |  |  |  |  | Impaired social functioning | self-constructed items (0.82) |  | WE: 0.12*  Drive: 0.26** | WE: β = 0.07  Drive: β = 0.02 |  |
|  |  |  |  |  |  |  |  | Co-worker support | Four items from the Job Content Questionnaire (0.72) |  | WE: −0.03  Drive: −0.15** | WE: β = −0.02  Drive: β = 0.00 |  |
|  |  |  |  |  |  |  |  | Supervisor support | Four items from the Job Content Questionnaire (0.82) |  | WE: −0.08*  Drive: 0.21** | WE: β = −0.00  Drive: β = −0.10* |  |
| 86 | Sharma & Sharma, 2018 | India | cross-sectional | 80 | n/a | 71 | DUWAS (n/a) | Work to family interference | Work-family Conflict Inventory  (Work to family interference (WFI): 0.81; Family to work Interference (FWI): 0.56) | Correlation,  Multiple regression analysis (outcome: WFI, FWI, and FSS) | with WFI:  WE: 0.53**  WC: 0.35**  DUWAS: 0.49**  with FWI:  WE: 0.34**  WC: 0.05  DUWAS: 0.23* | on WFI:  WE: β = 0.42**  WC: β = 0.09  DUWAS: β = 0.48**  on FWI:  WE: β = 0.48­­**  WC: β = 0.04  DUWAS: β = 0.23** |  |
|  |  |  |  |  |  |  |  | Family satisfaction | Family Satisfaction Scale (FSS) of Extra Work Satisfaction Scale (0.86) |  | WE: −0.28**  WC: −0.14  DUWAS: −0.23* | WE: β = −0.39**  WC: β = −0.14  DUWAS: β = −0.23** |  |
| 87 | Sheta & Hammouda, 2022 | Egypt | cross-sectional | 262 | n/a | 51.5 | WART (n/a) | social dysfunction | General Health Questionnaire (GHQ-28) (n/a) | ANOVA |  |  | No significant difference between WA (M = 7.55), medium WA (M = 7.25), and non-WA (M = 7.12) individuals. |
|  |  |  |  |  |  |  |  | quality of social relationships | WHO Quality of Life Scale (WHOQOL-BREF) (n/a) |  |  |  | non-WA (M = 7.12) individuals shoed higher quality of social relationships (M = 63.3) than WA (M = 61.12) and medium WA (M = 61.66) individuals.** |
| 88 | Shimazu & Schaufeli, 2009 | Japan | cross-sectional | 922 | 38.1 (12.8) | 93.8 | DUWAS  (WC: 0.70;  WE: 0.70) | Family satisfaction | One item (authors( (n/a) | Correlation | WE: −0.12**  WC: −0.11** |  |  |
| 89 | Shimazu et al., 2011 | Japan | cross-sectional | 2,992  (994 couples and their children) | Males:  37.9 (5.3)  Females:  36.1 (4.2 | 50 (among parents) | DUWAS  (WC: 0.72-0.75;  WE: 0.79-0.84) | Work-family conflict | Survey Work-Home Interaction – Nijmegen (SWING)  (Men 0.71; Women 0.85) | Correlation,  logistic regression analysis | Men WFC with:  Men WE: 0.59**  Men WC: 0.36**  Women WFC with  Women WE: 0.23**  Women WC: 0.33** |  |  |
|  |  |  |  |  |  |  |  | Family-Work conflict | Survey Work-Home Interaction – Nijmegen (SWING)  (Men 0.81; Women 0.78) |  | Women FWC with:  Men WE: –0.02  Men WC: 0.03  Men FWC with:  Women WE: 0.04  Women WC: 0.12** |  |  |
| 90 | Shimazu et al., 2014 | Japan | cross-sectional | 2,520 | 44.4 (12.9) | 49.9 | DUWAS  (WC:  Group1: 0.74; Group2: 0.72  WE:  Group1: 0.80;  Group2: 0.79) | Family/friend support | Brief Job Stress Questionnaire (BJSQ) (Group1: 0.86;  Group2: 0.85) | Correlation | WE: –0.08**  WC: –0.08** |  |  |
|  |  |  |  |  |  |  |  | Workplace support | Brief Job Stress Questionnaire (BJSQ) (Group1: 0.86;  Group2: 0.85) |  | WE: 0.02  WC: –0.03 |  |  |
| 91 | Shimazu et al., 2020 | Japan | cross-sectional | 208 families (Fathers: 208  Mothers:208) | Fathers: 39.7 (6.0)  Mothers: 38.1 (4.2) | 50 | DUWAS  (Fathers: 0.82; Mothers: 0.81) | Work-family conflict (WFC) | Survey Work-home Interaction-NijmeGen (SWING) (Fathers: 0.73; Mothers: 0.70) | Correlation,  SEM | Fathers: 0.52***  Mothers: 0.42*** | WFC mediated between work addiction and happiness among fathers, but not among mothers.  Indirect relationship of fathers’ workaholism with their child’s emotional and behavioral problems (β = 0.05*). It was not significant for mothers. |  |
|  |  |  |  |  |  |  |  | Work-family facilitation (WFF) | Survey Work-home Interaction-NijmeGen (SWING) (Fathers: 0.71; Mothers: 0.79) |  | Fathers: 0.21***  Mothers: 0.07 |  |  |
|  |  |  |  |  |  |  |  | Child’s Emotional and Behavioral Problem | Strength and Difficulties Questionnaire (SDQ) (0.67) |  | Fathers: –0.01  Mothers: –0.07 |  |  |
| 92 | Shin & Shin, 2020 | Korea | cross-sectional | 331 | n/a | 65.6 | Involvement and Drive subscales of WorkBat-R (0.83) (n/a) | Work-family conflict | 6 item scale by authors (n/a) (0.83) | Correlation, SEM (outcome: WFC) | 0.55** | β = 0.82**  Work addiction mediated between job security and WFC: β = 0.46** |  |
| 93 | Shkoler et al., 2017 | Israel | cross-sectional | Study 1: 158 | 33.80 (6.77) | 20.3 | Drive and Enjoyment subscales of the WorkBat  (Drive 0.73; Enjoyment 0.88) | Work-family conflict | Work-Family Conflict Scale (WFCS) (0.94) | Correlation, hierarchical regression (outcome: WFC) | Drive: 0.33**  Enjoyment: –0.32** | β = 0.26**  Interaction of Drive and job autonomy on WFC:  If job autonomy is low, the effect of Drive on WFC is near zero.  If job autonomy is moderate, the effect of Drive on WFC is positive (β = 0.21**).  If job autonomy is moderate, the effect of Drive on WFC is positive and strong (β = 0.47**).  WFC increase the relationship between Drive and Enjoyment. |  |
|  |  |  |  | Study 2: 349 | 27.04 (7.04) | 24.9 | Drive and Enjoyment subscales of the WorkBat  (Drive 0.73; Enjoyment 0.88) | Leader-Member Exchange | Leader-Member Exchange Questionnaire (LMX7) (0.85) | Correlation, hierarchical regression (outcome: WFC) | Drive: –0.03  Enjoyment: –0.07 | A higher LMX decrease the effect of Drive on Enjoyment.  A lower LMX increase the effect of Drive on Enjoyment. |  |
| 94 | Taheri et al., 2020 | Iran | cross-sectional | 414 | n/a | n/a | Workaholism Analysis Questionnaire (WAQ)  (WLC: 0.96;  WP: 0.92;  WA: 0.92;  U: 0.92;  WS: 0.90). | Work-family enrichment (WFE) | Work-Family Enrichment Scale (WFES) (0.96) | Correlation, SEM (outcome: WFE and FEW) | WLC: –0.36*  WP: –0.56*  WA: –0.40*  U: –0.60*  WS: –0.30* | β = –0.63** |  |
|  |  |  |  |  |  |  |  | Family-work enrichment (FEW) | Family-Work Enrichment Scale (0.96) |  | WLC: –0.06*  WP: –0.04*  WA: –0.06*  U: –0.13*  WS: –0.06* | β = –0.10 |  |
| 95 | Tahir & Aziz, 2019 | Pakistan | cross-sectional | 213 | n/a | 58.2 | WorkBat  (total: 0.93; Drive 0.39; Enjoyment 0.97; Involvement 0.49) | Work-family conflict | Work-Family Conflict Scale (WAFCS)  (0.97) | Correlation, linear regression (outcome: WFC) | 0.83** | β = 0.83** |  |
| 96 | Taylor et al., 2019 | USA | cross-sectional | 4,453 | 36 (11.1) | 54.5 | WART (0.81) | Work-family conflict (WFC) | Work-Family Conflict Scale (WFCS)  WFC: 0.92;  FWC: 0.89) | Correlation, SEM (outcome: WFC, FWC, Burnout) | 0.51* | WART → WFC: β = 0.63*  WART → WFC → Burnout: β = 0.22* |  |
|  |  |  |  |  |  |  |  | Family-work conflict (FWC) |  |  | 0.19* | WART → WFC β = 0.25*  WART → FWC → Burnout: β = –0.01 |  |
| 97 | Taylor et al., 2021 | USA | cross-sectional | 466 | 37.29 (10.32) | 49.4 | WART (0.82) | Work-family conflict (WFC) | Work-Family Conflict Scale (WFCS)  (WFC: 0.94;  FWC: 0.91) | Correlation, SEM (outcome: WFC, FWC, Burnout, Task coping, Emotion coping) | 0.59** | WART → WFC: β = 0.78**  WART → WFC → Emotion coping → Burnout: β = 0.08*  WART → WFC → Task coping → Burnout: β = 0.01* |  |
|  |  |  |  |  |  |  |  | Family-work conflict (FWC) |  |  | 0.17** | WART → FWC: β = 0.31**  WART → FWC → Emotion coping → Burnout: β = 0.01*  WART → FWC → Task coping → Burnout: β = 0.00* |  |
| 98 | Therthani et al., 2022 | USA | cross-sectional | 106 | 44.72 (11.34) | 39 | WAQ (0.92) | quality of relationships | Life Balance Inventory (LBI)  ( from 0.74 (substance use) to 0.91 (positive orientation)) | Correlation,  multiple regression analysis (outcome: WA) | –0.16 | β = –0.09 |  |
|  |  |  |  |  |  |  |  | friendship |  |  | –0.45** | β = –0.21** |  |
|  |  |  |  |  |  |  |  | sex/intimacy |  |  | –0.22** | β = –0.01 |  |
| 99 | Torp et al., 2018 | Norway | cross-sectional | 2,186 | n/a | 61 | DUWAS (0.86) | Work-family conflict | Work-Family Conflict Scale (WFCS) (0.81) | Correlation, multiple regression analysis (outcome: WFC) | 0.57** | β = 0.30*  Role overload mediated the effect of work addiction on WFC. |  |
|  |  |  |  |  |  |  |  | Social support from supervisors | items from the Questionnaire of the Knowledge Intensive Work Environment Study (0.88) |  | –0.13** |  |  |
|  |  |  |  |  |  |  |  | Cohesion in work teams | items from the Questionnaire of the Knowledge Intensive Work Environment Study (0.75) |  | –0.21** |  |  |
|  |  |  |  |  |  |  |  | Social community at work | items from the Questionnaire of the Knowledge Intensive Work Environment Study (0.84) |  | –0.20** |  |  |
| 100 | Vedoato et al., 2021 | Brazilia | cross-sectional | 333 | (n/a) | 12.3 | DUWAS-10 (n/a) | Quality social relationships (QSR) | WHO Quality of Life Assessment Instrument-Bref (WHOQOL-Bref) (n/a) | Multiple logistic regression (outcome: WE, WC) |  | WE predicted by QSR:  OR = 0.28**  WC predicted by QRS:  OR = 0.39** |  |
| 101 | Xie et al., 2022 | China | cross-sectional | 403 | 32.25 (6.34) | 1.2 | DUWAS-10 (0.82) | Leader-member exchange | Leader-Member Exchange scale (0.84) | Correlation, path analysis (outcome: WA) | –0.26** | β = −0.20**  the interaction between LMX and role overload predicts WA:  β = −0.10* |  |
| 102 | Xu & Li, 2021 | China | cross-sectional | 425 | 29.9 (7.08) | 40.7 | MWS (Motivational: 0.82,  Cognitive: 0.89; Emotional: 0.89; Behavioral: 0.87) | Work-family conflict | Work Family Conflict Scale (0.84) | Correlation | Motivational: 0.26**  Cognitive: 0.39**  Emotional: 0.35**  Behavioral: 0.33** |  |  |

Note. Abbreviations for variables: Behav., behavioral; Cogn., cognitive; CoNWP, children of non-workaholic parents; CoWP, children of workaholic parents; CT, compulsive tendencies; Deleg., inability to delegate; DRM, day reconstruction method; Emot., emotional; Emot/Perf., Emotional arousal/perfectionism; FEW, family-work enrichment; FIW, family interference with work; FLS, family life satisfaction; FSSB, family-supportive supervisor behavior; FWC, family-work conflict; FWE, family work enrichment, FWI, family to work interference; HWC, home-work conflict, HWE, home-work enrichment, IC, impaired communication, JD, job demands, LMX, leader-member exchange; Motiv., motivational; NC, need for control; NHWI, negative home–work interference, Nwcs, non-workaholics, NWHI, negative work–home interference, Obs/Comp., obsession/compulsion; OR, time communicating with significant others - ratings of significant others; Overd., overdoing; PLIW, personal life interference with work; QSR, quality social relationships; Res., result orientation; SDQ, strength and Difficulties Questionnaire; Self, self-worth; SP, social pessimism; SR, time communicating with significant others – self-ratings; SW, self-worth; U, unpleasantness; W, work addiction; WC, working compulsively; Wcs, workaholics; WE, working excessively; WFC, work-family conflict; WFE, work-family enrichment; WFF, work-family facilitation; WFI, work to family interference; WHC, work-home conflict; WHE, work-home enrichment; WIF, work interference with family; WIPL, work interference with personal life; WLB, work-life balance; WLC, work-life conflict; WLF, work-life fit; WNC, work-nonwork conﬂict; WNE, work–nonwork enrichment; WP, work perfectionism; WS, withdrawal symptoms.

Abbreviations for work addiction scales: BWAS, Bergen Work Addiction Scale; CWST, Children of Workaholic Parents Screening Test; DUWAS, Dutch Work Addiction Scale; MWAQ, Multidimensional Workaholism Assessment Questionnaire; MWS, Multidimensional Workaholism Scale; SZAP, Excessive Work Involvement Scale, SNAP, Schedule for Adaptive and Nonadaptive Personality; SWBT, Scale of Workaholism as Behavioral Tendencies; WAQ, Workaholism Analysis Questionnaire; WART, Work Addiction Risk Test; WorkBat, Workaholism Battery; Work-related Inventory (WI-10).

Abbreviations for social relationship measures: BJSQ, Job Stress Questionnaire; BSSS, Berlin Social Support Scale; DAS, Dyadic Adjustment Scale; FAD, McMaster Family Assessment Device; FWES, Family-Work Enrichment Scale; FOS, Family of Origin Scale; FSSB-SF, Family Supportive Supervisor Behavior Short-Form; GHQ-28, General Health Questionnaire; HSE Indicator Tool, Health and Safety Executive Indicator Tool; JBLI, Juhnke–Balkin Life Balance Inventory; KMSS, Kansas Marital Satisfaction Scale; MSPSS, Multidimensional Scale of Perceived Social Support; SAQ, Strategy and Attribution Questionnaire; SF-36, Rand Short Form-36; SPOS, Survey of Perceived Organizational Support; SWING, Survey Work– Home Interference NijmeGen; WFCS, Work Family Conflict Scale; WFES, Work and Family Engagement Survey; WFIS, Work-Family Interface Scale, WIF, work interference with family scale; WLBS, Work-Life Balance Scale; WLIB, Work-Life Imbalance Scale; WIPLS, Work Interference with Personally Life Scale; WFES, Work-Family Enrichment Scale; WHOQOL-Bref, World Health Organization Quality of Life Assessment Instrument-Bref.

* p < 0.05; ** p < 0.01; ***p < 0.001; n.s., non-significant result

**References**

Andreassen, C. S., Griffiths, M. D., Gjertsen, S. R., Krossbakken, E., Kvam, S., & Pallesen, S. (2013). The relationships between behavioral addictions and the five-factor model of personality. *Journal of Behavioral Addictions*, *2*(2), 90–99. https://doi.org/10.1556/jba.2.2013.003

Ayar, D., Karaman, M. A., & Karaman, R. (2022). Work-Life Balance and Mental Health Needs of Health Professionals During COVID-19 Pandemic in Turkey. *International Journal of Mental Health and Addiction*, *20*(1), 639–655. https://doi.org/10.1007/s11469-021-00717-6

Aziz, S., Adkins, C. T., Walker, A. G., & Wuensch, K. L. (2010). Workaholism and work-life imbalance: Does cultural origin influence the relationship? *International Journal of Psychology*, *45*(1), 72–79. https://doi.org/10.1080/00207590902913442

Aziz, S., & Cunningham, J. (2008). Workaholism, work stress, work‐life imbalance: Exploring gender’s role. *Gender in Management: An International Journal*, *23*(8), 553–566. https://doi.org/10.1108/17542410810912681

Aziz, S., Wuensch, K. L., & Brandon, H. R. (2010). A comparison among worker types using a composites approach and median splits. *The Psychological Record*, *60*(4), 627–642.

Aziz, S., & Zickar, M. J. (2006). A cluster analysis investigation of workaholism as a syndrome. *Journal of Occupational Health Psychology*, *11*(1), 52–62. https://doi.org/10.1037/1076-8998.11.1.52

B. Bakker, A., Shimazu, A., Demerouti, E., Shimada, K., & Kawakami, N. (2014). Work engagement versus workaholism: A test of the spillover-crossover model. *Journal of Managerial Psychology*, *29*(1), 63–80. https://doi.org/10.1108/JMP-05-2013-0148

Babic, A., Stinglhamber, F., Barbier, M., & Hansez, I. (2022). Work environment and work-to-family conflict: Examining the mediating role of heavy work investment. *Journal of Management & Organization*, *28*(2), 398–421. https://doi.org/10.1017/jmo.2019.40

Bakker, A. B., Demerouti, E., & Burke, R. (2009). Workaholism and relationship quality: A spillover-crossover perspective. *Journal of Occupational Health Psychology*, *14*(1), 23–33. https://doi.org/10.1037/a0013290

Bakker, A. B., Demerouti, E., Oerlemans, W., & Sonnentag, S. (2013). Workaholism and daily recovery: A day reconstruction study of leisure activities. *Journal of Organizational Behavior*, *34*(1), 87–107. https://doi.org/10.1002/job.1796

Balducci, C., Avanzi, L., Consiglio, C., Fraccaroli, F., & Schaufeli, W. (2017). A cross-national study on the psychometric quality of the Italian version of the Dutch Work Addiction Scale (DUWAS). *European Journal of Psychological Assessment*, *33*(6), 422–428. https://doi.org/10.1027/1015-5759/a000300

Balkin, R. S., Reiner, S. M., Hendricks, L., Washington, A., McNeary, S., Juhnke, G. A., & Hunter, Q. (2018). Life balance and work addiction among African Americans. *The Career Development Quarterly*, *66*(1), 77–84. https://doi.org/10.1002/cdq.12123

Bartczak, M., & Ogińska-Bulik, N. (2012). Workaholism and mental health among Polish academic workers. *International Journal of Occupational Safety and Ergonomics: JOSE*, *18*(1), 3–13. https://doi.org/10.1080/10803548.2012.11076910

Bayhan Karapinar, P., Metin Camgoz, S., & Tayfur Ekmekci, O. (2020). Employee Wellbeing, Workaholism, Work–Family Conflict and Instrumental Spousal Support: A Moderated Mediation Model. *Journal of Happiness Studies*, *21*(7), 2451–2471. https://doi.org/10.1007/s10902-019-00191-x

Bonebright, C. A., Clay, D. L., & Ankenmann, R. D. (2000). The relationship of workaholism with work–life conflict, life satisfaction, and purpose in life. *Journal of Counseling Psychology*, *47*(4), 469–477. https://doi.org/10.1037/0022-0167.47.4.469

Borges, E. M. das N., Sequeira, C. A. da C., Queirós, C. M. L., & Mosteiro-Díaz, M. P. (2021). Workaholism and family interaction among nurses. *Ciencia & Saude Coletiva*, *26*(12), 5945–5953. https://doi.org/10.1590/1413-812320212612.13842021

Brady, B. R., Vodanovich, S. J., & Rotunda, R. (2008). The impact of workaholism on work-family conflict, job satisfaction, and perception of leisure activities. *The Psychologist-Manager Journal*, *11*(2), 241–263. https://doi.org/10.1080/10887150802371781

Braun, A. C., de Lara Machado, W., Luiz de Andrade, A., & Oliveira, M. Z. de. (2019). Why work-family conflict can drive your executives away? *Revista de Psicología*, *37*(1), 251–278.

Burke, R. J. (1999). Workaholism and extra-work satisfactions. *The International Journal of Organizational Analysis*, *7*(4), 352–364. https://doi.org/10.1108/eb028906

Burke, R. J. (2000). Workaholism and divorce. *Psychological Reports*, *86*(1), 219–220. https://doi.org/10.2466/PR0.86.1.219-220

Burke, R. J., Burgess, Z., & Oberklaid, F. (2003). Workaholism and Divorce among Australian Psychologists. *Psychological Reports*, *93*(1), 91–92. https://doi.org/10.2466/pr0.2003.93.1.91

Burke, R. J., & Fiksenbaum, L. (2009). Work Motivations, Work Outcomes, and Health: Passion Versus Addiction. *Journal of Business Ethics*, *84*(2), 257–263. https://doi.org/10.1007/s10551-008-9697-0

Caesens, G., Stinglhamber, F., & Luypaert, G. (2014). The impact of work engagement and workaholism on well-being: The role of work-related social support. *Career Development International*, *19*(7), 813–835. https://doi.org/10.1108/CDI-09-2013-0114

Chamberlin, C. M., & Zhang, N. (2009). Workaholism, health, and self-acceptance. *Journal of Counseling & Development*, *87*(2), 159–169. https://doi.org/10.1002/j.1556-6678.2009.tb00563.x

Chang, P.-C., Gao, X., Wu, T., & Lin, Y.-Y. (2022). Workaholism and work–family conflict: A moderated mediation model of psychological detachment from work and family-supportive supervisor behavior. *Chinese Management Studies*, *17*(4), 770–786. https://doi.org/10.1108/CMS-09-2021-0380

Clark, M. A., Michel, J. S., Stevens, G. W., Howell, J. W., & Scruggs, R. S. (2014). Workaholism, work engagement and work-home outcomes: Exploring the mediating role of positive and negative emotions. *Stress and Health: Journal of the International Society for the Investigation of Stress*, *30*(4), 287–300. https://doi.org/10.1002/smi.2511

Converso, D., Sottimano, I., Molinengo, G., & Loera, B. (2019). The Unbearable Lightness of the Academic Work: The Positive and Negative Sides of Heavy Work Investment in a Sample of Italian University Professors and Researchers. *Sustainability*, *11*(8), Article 8. https://doi.org/10.3390/su11082439

Daniel, C., Gentina, E., & Mesmer-Magnus, J. (2022). Mindfulness buffers the deleterious effects of workaholism for work-family conflict. *Social Science & Medicine*, *306*, 115118. https://doi.org/10.1016/j.socscimed.2022.115118

Del Líbano, M., Llorens, S., Salanoval, M., & Schaufeli, W. B. (2012). About the dark and bright sides of self-efficacy: Workaholism and work engagement. *The Spanish Journal of Psychology*, *15*(2), 688–701. https://doi.org/10.5209/rev_sjop.2012.v15.n2.38883

Di Stefano, G., & Gaudiino, M. (2018). Differential Effects of Workaholism and Work Engagement on the Interference Between Life and Work Domains. *Europe’s Journal of Psychology*, *14*(4), 863–879. https://doi.org/10.5964/ejop.v14i4.1626

Dong, M., Zhang, T., Li, Y., & Ren, Z. (2022). The Effect of Work Connectivity Behavior After-Hours on Employee Psychological Distress: The Role of Leader Workaholism and Work-to-Family Conflict. *Frontiers in Public Health*, *10*, 722679. https://doi.org/10.3389/fpubh.2022.722679

Eason, C. M., Gilgallon, T. J., & Singe, S. M. (2022). Work-Addiction Risk in Athletic Trainers and Its Relationship to Work-Family Conflict and Burnout. *Journal of Athletic Training*, *57*(3), 225–233. https://doi.org/10.4085/JAT0348-20

Falco, A., Girardi, D., De Carlo, A., Andreassen, C. S., & Dal Corso, L. (2022). Work Addiction among Bank Employees in Italy: A Contribution to Validation of the Bergen Work Addiction Scale with a Focus on Measurement Invariance across Gender and Managerial Status. *Sustainability*, *14*(21), Article 21. https://doi.org/10.3390/su142113714

Falco, A., Kravina, L., Girardi, D., Dal Corso, L., Di Sipio, A., & De Carlo, N. A. (2012). The convergence between self and observer ratings of workaholism: A comparison between couples. *TPM-Testing, Psychometrics, Methodology in Applied Psychology*, *19*(4), 311–324.

Flowers, C., Robinson, B. E., & Carroll, J. J. (2000). Criterion-related validity of the Marital Disaffection Scale as a measure of marital estrangement. *Psychological Reports*, *86*(3 Pt 2), 1101–1103. https://doi.org/10.2466/pr0.2000.86.3c.1101

Fujiwara, T., Shimazu, A., Tokita, M., Shimada, K., Takahashi, M., Watai, I., Iwata, N., & Kawakami, N. (2016). Association between Parental Workaholism and Body Mass Index of Offspring: A Prospective Study among Japanese Dual Workers. *Frontiers in Public Health*, *4*, 41. https://doi.org/10.3389/fpubh.2016.00041

Gillet, N., Austin, S., Fernet, C., Sandrin, E., Lorho, F., Brault, S., Becker, M., & Aubouin Bonnaventure, J. (2021). Workaholism, presenteeism, work-family conflicts and personal and work outcomes: Testing a moderated mediation model. *Journal of Clinical Nursing*, *30*(19–20), 2842–2853. https://doi.org/10.1111/jocn.15791

Gillet, N., Morin, A. J. S., Cougot, B., & Gagné, M. (2017). Workaholism profiles: Associations with determinants, correlates, and outcomes. *Journal of Occupational and Organizational Psychology*, *90*(4), 559–586. https://doi.org/10.1111/joop.12185

Gillet, N., Morin, A. J. S., Ndiaye, A., Colombat, P., Sandrin, E., & Fouquereau, E. (2022). Complementary variable- and person-centred approaches to the dimensionality of workaholism. *Applied Psychology*, *71*(1), 312–355. https://doi.org/10.1111/apps.12323

Gillet, N., Morin, A. J. S., Sandrin, E., & Houle, S. A. (2018). Investigating the combined effects of workaholism and work engagement: A substantive-methodological synergy of variable-centered and person-centered methodologies. *Journal of Vocational Behavior*, *109*, 54–77. https://doi.org/10.1016/j.jvb.2018.09.006

Grawitch, M. J., Werth, P. M., Palmer, S. N., Erb, K. R., & Lavigne, K. N. (2018). Self-imposed pressure or organizational norms? Further examination of the construct of workplace telepressure. *Stress and Health: Journal of the International Society for the Investigation of Stress*, *34*(2), 306–319. https://doi.org/10.1002/smi.2792

Hakanen, J., & Peeters, M. (2015). How Do Work Engagement, Workaholism, and the Work-to-Family Interface Affect Each Other? A 7-Year Follow-Up Study. *Journal of Occupational and Environmental Medicine*, *57*(6), 601–609. https://doi.org/10.1097/JOM.0000000000000457

Hamilton Skurak, H., Malinen, S., Näswall, K., & Kuntz, J. C. (2021). Employee wellbeing: The role of psychological detachment on the relationship between engagement and work–life conflict. *Economic and Industrial Democracy*, *42*(1), 116–141. https://doi.org/10.1177/0143831X17750473

Hancock, M. G., Balkin, R. S., Reiner, S. M., Williams, S., Hunter, Q., Powell, B., & Juhnke, G. A. (2019). Life balance and work addiction among NCAA administrators and coaches. *The Career Development Quarterly*, *67*(3), 264–270. https://doi.org/10.1002/cdq.12195

Hauk, M., & Chodkiewicz, J. (2013). The role of general and occupational stress in the relationship between workaholism and work-family/family-work conflicts. *International Journal of Occupational Medicine and Environmental Health*, *26*(3), 383–393. https://doi.org/10.2478/s13382-013-0087-1

Hirschi, A., Keller, A. C., & Spurk, D. (2019). Calling as a double-edged sword for work-nonwork enrichment and conflict among older workers. *Journal of Vocational Behavior*, *114*, 100–111. https://doi.org/10.1016/j.jvb.2019.02.004

Hogan, V., Hogan, M., & Hodgins, M. (2016). A study of workaholism in Irish academics. *Occupational Medicine (Oxford, England)*, *66*(6), 460–465. https://doi.org/10.1093/occmed/kqw032

Huml, M. R., Taylor, E. A., & Dixon, M. A. (2021). From engaged worker to workaholic: A mediated model of athletic department employees. *European Sport Management Quarterly*, *21*(4), 583–604. https://doi.org/10.1080/16184742.2020.1765404

Huyghebaert-Zouaghi, T., Caesens, G., Sandrin, É., & Gillet, N. (2022). Workaholism and work engagement: An examination of their psychometric multidimensionality and relations with employees’ functioning. *Current Psychology*, *42*(7), 5240–5253. https://doi.org/10.1007/s12144-021-01820-6

Innanen, H., Tolvanen, A., & Salmela-Aro, K. (2014). Burnout, work engagement and workaholism among highly educated employees: Profiles, antecedents and outcomes. *Burnout Research*, *1*(1), 38–49. https://doi.org/10.1016/j.burn.2014.04.001

Kasemy, Z. A., Abd-Ellatif, E. E., Abdel Latif, A. A., Bahgat, N. M., Shereda, H. M. A., Shattla, S. I., Aboalizm, S. E., Abd Elhy, A. H., Allam, A. R., Ramadan, A. N., Amer, H. M., Ahmed, N. A., AlJifri, A. A., & El Dalatony, M. M. (2020). Prevalence of Workaholism Among Egyptian Healthcare Workers With Assessment of Its Relation to Quality of Life, Mental Health and Burnout. *Frontiers in Public Health*, *8*. https://www.frontiersin.org/articles/10.3389/fpubh.2020.581373

Kim, S., Jeong, W., Jang, S.-I., Park, E.-C., & Park, S. (2021). Is Work Hour Mismatch Associated with Depression? *Safety and Health at Work*, *12*(1), 96–101. https://doi.org/10.1016/j.shaw.2020.09.009

Kochanska, G., Friesenborg, A. E., Lange, L. A., Martel, M. M., & Kochanska, G. (2004). Parents’ personality and infants’ temperament as contributors to their emerging relationship. *Journal of Personality and Social Psychology*, *86*(5), 744–759. https://doi.org/10.1037/0022-3514.86.5.744

Kravina, L., Falco, A., De Carlo, N. A., Andreassen, C. S., & Pallesen, S. (2014). Workaholism and work engagement in the family: The relationship between parents and children as a risk factor. *European Journal of Work and Organizational Psychology*, *23*(6), 875–883. https://doi.org/10.1080/1359432X.2013.832208

Kravina, L., Falco, A., Girardi, D., & De Carlo, N. A. (2010). Workaholism among management and workers in an Italian cooperative enterprise. *TPM-Testing, Psychometrics, Methodology in Applied Psychology*, *17*, 201–216.

Lanaj, K., Gabriel, A. S., & Chawla, N. (2021). The self-sacrificial nature of leader identity: Understanding the costs and benefits at work and home. *The Journal of Applied Psychology*, *106*(3), 345–363. https://doi.org/10.1037/apl0000505

Levy, D. V. (2015a). Effects of workaholism on satisfaction among US managerial and professional women. *Gender in Management: An International Journal*, *30*(8), 635–651. https://doi.org/10.1108/GM-08-2014-0076

Levy, D. V. (2015b). Workaholism and Marital Satisfaction Among Female Professionals. *The Family Journal*, *23*(4), 330–335. https://doi.org/10.1177/1066480715601109

Loscalzo, Y. (2021). The Impact of Workaholism and Work Engagement on Distant Learning and Work-Family Conflict During the COVID-19 Lockdown. *The Amfiteatru Economic Journal*, *23*(58). https://econpapers.repec.org/article/aesamfeco/v_3a23_3ay_3a2021_3ai_3a58_3ap_3a752.htm

Lundkvist, E., Gustafsson, H., Davis, P., & Hassmén, P. (2016). Workaholism, Home–Work/Work–Home Interference, and Exhaustion Among Sports Coaches. *Journal of Clinical Sport Psychology*, *10*(3), 222–236. https://doi.org/10.1123/jcsp.2015-0029

Mazzetti, G., Vignoli, M., Schaufeli, W. B., & Guglielmi, D. (2019). Work addiction and presenteeism: The buffering role of managerial support. *International Journal of Psychology: Journal International De Psychologie*, *54*(2), 174–179. https://doi.org/10.1002/ijop.12449

McMillan, L. H. W., & O’Driscoll, M. P. (2004). Workaholism and health: Implications for organizations. *Journal of Organizational Change Management*, *17*(5), 509–519. https://doi.org/10.1108/09534810410554515

Mcmillan, L. H. W., O’Driscoll, M. P., & Brady, E. C. (2004). The impact of workaholism on personal relationships. *British Journal of Guidance & Counselling*, *32*(2), 171–186. https://doi.org/10.1080/03069880410001697729

Molino, M., Bakker, A. B., & Ghislieri, C. (2016). The role of workaholism in the job demands-resources model. *Anxiety, Stress, and Coping*, *29*(4), 400–414. https://doi.org/10.1080/10615806.2015.1070833

Molino, M., Kovalchuk, L. S., Ghislieri, C., & Spagnoli, P. (2022). Work Addiction Among Employees and Self-Employed Workers: An Investigation Based on the Italian Version of the Bergen Work Addiction Scale. *Europe’s Journal of Psychology*, *18*(3), Article 3. https://doi.org/10.5964/ejop.2607

Morkevičiūtė, M., & Endriulaitienė, A. (2022). Understanding Work Addiction in Adult Children: The Effect of Addicted Parents and Work Motivation. *International Journal of Environmental Research and Public Health*, *19*(18), 11279. https://doi.org/10.3390/ijerph191811279

Niehuis, S. (2007). Convergent and discriminant validity of the Marital Disillusionment Scale. *Psychological Reports*, *100*(1), 203–207. https://doi.org/10.2466/pr0.100.1.203-207

Omar, A., Salessi, S., Vaamonde, J. D., & Urteaga, F. (2021). Psychometric qualities of the Argentine version of the Dutch work addiction scale (DUWAS). *Current Psychology*, *40*(2), 793–803. https://doi.org/10.1007/s12144-018-9999-z

Peplińska, A., Wojdylo, K., Kosakowska-Berezecka, N., & Połomski, P. (2015). The role of purpose in life and social support in reducing the risk of workaholism among women in Poland. *Health Psychology Report*, *3*(4), 326–335. https://doi.org/10.5114/hpr.2015.50902

Quiñones-García, C., & Korak-Kakabadse, N. (2014). Compulsive internet use in adults: A study of prevalence and drivers within the current economic climate in the UK. *Computers in Human Behavior*, *30*, 171–180. https://doi.org/10.1016/j.chb.2013.08.004

Rai, A., Kim, M., & Beehr, T. A. (2022). Does competitive climate at work lead to problems at home? Examination of mediating pathways. *Asia Pacific Journal of Human Resources*, *n/a*(n/a). https://doi.org/10.1111/1744-7941.12362

Reiner, S. M., Balkin, R. S., Gotham, K. R., Hunter, Q., Juhnke, G. A., & Davis, R. J. (2019). Assessing life balance and work addiction in high‐pressure, high‐demand careers. *Journal of Counseling & Development*, *97*(4), 409–416. https://doi.org/10.1002/jcad.12289

Robinson, B. E., & Carroll, J. J. (1999). Assessing the offspring of workaholic parents: The Children of Workaholics Screening Test. *Perceptual and Motor Skills*, *88*(3 Pt 2), 1127–1134. https://doi.org/10.2466/pms.1999.88.3c.1127

Robinson, B. E., Carroll, J. J., & Flowers, C. (2001). Marital estrangement, positive affect and locus of control among spouses of workaholics and spouses of nonworkaholics: A national study. *American Journal of Family Therapy*, *29*(5), 397–410. https://doi.org/10.1080/01926180127624

Robinson, B. E., Flowers, C., & Carroll, J. (2001). Work Stress and Marriage: A Theoretical Model Examining the Relationship Between Workaholism and Marital Cohesion. *International Journal of Stress Management*, *8*(2), 165–175. https://doi.org/10.1023/A:1009533415030

Robinson, B. E., Flowers, C., & Ng, K.-M. (2006). The Relationship Between Workaholism and Marital Disaffection: Husbands’ Perspective. *The Family Journal*, *14*(3), 213–220. https://doi.org/10.1177/1066480706287269

Robinson, B. E., & Kelley, L. (1998). Adult children of workaholics: Self-concept, anxiety, depression, and locus of control. *American Journal of Family Therapy*, *26*(3), 223–238. https://doi.org/10.1080/01926189808251102

Robinson, B. E., & Kelley, L. (1999). School Age Workaholic Children: Type A Behaviors, Self-esteem, Anxiety and Locus of Control. *Early Child Development and Care*, *158*(1), 43–50. https://doi.org/10.1080/0300443991580105

Robinson, B. E., & Post, P. (1995). Work Addiction as a Function of Family of Origin and Its Influence on Current Family Functioning. *The Family Journal*, *3*(3), 200–206. https://doi.org/10.1177/1066480795033003

Robinson, B. E., & Post, P. (1997). Risk of addiction to work and family functioning. *Psychological Reports*, *81*(1), 91–95. https://doi.org/10.2466/PR0.81.5.91-95

Ruiz-Garcia, P., Castanheira, A. M., Borges, E., & Mosteiro-Diaz, M.-P. (2022). Workaholism and work-family interaction among emergency and critical care nurses. *Intensive and Critical Care Nursing*, *72*, 103240. https://doi.org/10.1016/j.iccn.2022.103240

Russo, J. A., & Waters, L. E. (2006). Workaholic worker type differences in work‐family conflict: The moderating role of supervisor support and flexible work scheduling. *Career Development International*, *11*(5), 418–439. https://doi.org/10.1108/13620430610683052

Sawhney, G., Delongchamp, A., Sinclair, R. R., & Britt, T. W. (2022). Daily expression of workaholism and family outcomes: The buffering and magnifying effects of economic resources. *Stress and Health: Journal of the International Society for the Investigation of Stress*, *39*(1), 74–86. https://doi.org/10.1002/smi.3169

Scafuri Kovalchuk, L., Buono, C., Ingusci, E., Maiorano, F., De Carlo, E., Madaro, A., & Spagnoli, P. (2019). Can Work Engagement Be a Resource for Reducing Workaholism’s Undesirable Outcomes? A Multiple Mediating Model Including Moderated Mediation Analysis. *International Journal of Environmental Research and Public Health*, *16*(8), 1402. https://doi.org/10.3390/ijerph16081402

Schaufeli, W. B., Taris, T. W., & van Rhenen, W. (2008). Workaholism, burnout, and work engagement: Three of a kind or three different kinds of employee well-being? *Applied Psychology: An International Review*, *57*(2), 173–203. https://doi.org/10.1111/j.1464-0597.2007.00285.x

Sharma, J., & Sharma, P. (2018). Workaholism and bank employees’ well-being: An inside look. *International Journal of Business Excellence*, *15*(3), 289–307. https://doi.org/10.1504/IJBEX.2018.092572

Sheta, S. S., & Hammouda, M. A. (2022). Risk for Workaholism among Working Physicians of Zagazig University Hospitals: A Massage for Achieving Productive Work and Balanced Life. *The Egyptian Journal of Hospital Medicine*, *89*(1), 4402–4409. https://doi.org/10.21608/ejhm.2022.258451

Shimazu, A., Bakker, A. B., Demerouti, E., Fujiwara, T., Iwata, N., Shimada, K., Takahashi, M., Tokita, M., Watai, I., & Kawakami, N. (2020). Workaholism, Work Engagement and Child Well-Being: A Test of the Spillover-Crossover Model. *International Journal of Environmental Research and Public Health*, *17*(17), 6213. https://doi.org/10.3390/ijerph17176213

Shimazu, A., de Jonge, J., Kubota, K., & Kawakami, N. (2014). Psychological detachment from work during off-job time: Predictive role of work and non-work factors in Japanese employees. *Industrial Health*, *52*(2), 141–146. https://doi.org/10.2486/indhealth.2013-0210

Shimazu, A., Demerouti, E., Bakker, A. B., Shimada, K., & Kawakami, N. (2011). Workaholism and well-being among Japanese dual-earner couples: A spillover-crossover perspective. *Social Science & Medicine (1982)*, *73*(3), 399–409. https://doi.org/10.1016/j.socscimed.2011.05.049

Shimazu, A., & Schaufeli, W. B. (2009). Is Workaholism Good or Bad for Employee Well-being? The Distinctiveness of Workaholism and Work Engagement among Japanese Employees. *Industrial Health*, *47*(5), 495–502. https://doi.org/10.2486/indhealth.47.495

Shin, J., & Shin, H. (2020). Impact of Job Insecurity on Hotel Workers’ Workaholism and Work–Family Conflict in Korea. *International Journal of Environmental Research and Public Health*, *17*(21), 7783. https://doi.org/10.3390/ijerph17217783

Shkoler, O., Rabenu, E., & Tziner, A. (2017). The dimensionality of workaholism and its relations with internal and external factors. *Revista de Psicología Del Trabajo y de Las Organizaciones*, *33*(3), 193–203. https://doi.org/10.1016/j.rpto.2017.09.002

Taheri, F., Asarian, M., & Shahhosseini, P. (2020). Workaholism and workplace incivility: The role of work–family enrichment. *Management Decision*, *59*(2), 372–389. https://doi.org/10.1108/MD-08-2019-1035

Tahir, S., & Aziz, S. (2019). *Workaholism as predictor of work-family conflict and mental well-being of public and private sector employees*. https://www.semanticscholar.org/paper/Workaholism-as-predictor-of-work-family-conflict-of-Tahir-Aziz/df6807b08f2bca3c8ff59bedfe13b2bd9c3fa21f

Taylor, E. A., Huml, M. R., & Dixon, M. A. (2019). Workaholism in Sport: A Mediated Model of Work–Family Conflict and Burnout. *Journal of Sport Management*, *33*(4), 249–260. https://doi.org/10.1123/jsm.2018-0248

Taylor, E., Huml, M., Cohen, A., & Lopez, C. (2021). The Impacts of Work–Family Interface and Coping Strategy on the Relationship between Workaholism and Burnout in Campus Recreation and Leisure Employees. *Leisure Studies*, *40*(5), 714–729. https://doi.org/10.1080/02614367.2021.1879908

Therthani, S., Balkin, R. S., Perepiczka, M., Silva, S., Hunter, Q., & Juhnke, G. A. (2022). Assessing personality traits, life balance domains, and work addiction among entrepreneurs. *The Career Development Quarterly*, *70*(3), 190–201. https://doi.org/10.1002/cdq.12296

Torp, S., Lysfjord, L., & Midje, H. H. (2018). Workaholism and work–family conflict among university academics. *Higher Education*, *76*(6), 1071–1090. https://doi.org/10.1007/s10734-018-0247-0

Vedoato, T., Pedro, D. R. C., Galdino, M. J. Q., Aroni, P., Radovanovic, C. A. T., Martins, J. T., & Haddad, M. do C. F. L. (2021). Association between workaholism and quality of life in stricto sensu graduate professors in nursing. *Revista Brasileira De Enfermagem*, *74*(2), e20190901. https://doi.org/10.1590/0034-7167-2019-0901

Xie, L.-L., Jiang, W., Niyomsilp, E., Jing, J., Feng, L., Wen, Y., Wang, L., & Zheng, R. (2022). Effect of role overload on missed nursing care in China: The role of work addiction and leader-member exchange. *Nursing Open*, *10*(5), 3153–3163. https://doi.org/10.1002/nop2.1565

Xu, Y., & Li, C. (2021). Validation of the Chinese Version of the Multidimensional Workaholism Scale. *Journal of Career Assessment*, *29*(4), 608–623. https://doi.org/10.1177/1069072721994272
