# Supplementary material for: Work addiction and social functioning: A systematic review and five meta-analyses
Source: PLoS One. 2024 Jun 4;19(6):e0303563. doi: 10.1371/journal.pone.0303563 (PMC11149883; doi:10.1371/journal.pone.0303563)
Supplement: S2 Table — (DOCX) [file pone.0303563.s006.docx]

**S2 Table. Quality analysis of the primary articles included in the systematic review.**

|  | **Study** | **JBI Critical Appraisal Checklist** | | | | | | | | | |
| --- | --- | --- | --- | --- | --- | --- | --- | --- | --- | --- | --- |
|  |  | **1.**  **Inclusion** | **2.**  **Study description** | **3.**  **Exposure** | **4.**  **Condition measurement** | **5.**  **Confounds identified** | **6.**  **Strategies for confounds** | **7.**  **Outcome measurement** | **8. Statistical analysis** | **Total**  **score** | **Total score (%)** |
| 1 | Andreassen et al., 2013 (27) | Yes | Yes | N/A | Yes | Yes | Yes | Yes | Yes | 7 | 100% |
| 2 | Ayar et al., 2021 (28) | Yes | Yes | N/A | Yes | Yes | Yes | Yes | Yes | 7 | 100% |
| 5 | Aziz, Adkins et al., 2010 (29) | Yes | No | N/A | Yes | Yes | Yes | Yes | Yes | 6 | 86% |
| 4 | Aziz & Cunnigham, 2008 (30) | Yes | No | N/A | Yes | No | No | Yes | Yes | 4 | 57.1% |
| 6 | Aziz, Wuensch et al., 2010 (31) | Yes | No | N/A | Yes | Yes | Yes | Yes | Yes | 6 | 86% |
| 3 | Aziz & Zickar, 2006 (32) | Yes | No | N/A | Yes | Yes | Yes | Yes | Yes | 5 | 71.4% |
| 7 | Babic et al., 2022 (33) | Yes | No | N/A | Yes | Yes | Yes | Yes | Yes | 6 | 86% |
| 8 | Bakker et al., 2009 (34) | Yes | Yes | N/A | Yes | Yes | Yes | Yes | Yes | 7 | 100% |
| 9 | Bakker et al., 2013 (35) | Yes | Yes | N/A | Yes | Yes | Yes | Yes | Yes | 7 | 100% |
| 10 | Bakker et al., 2014 (36) | Yes | Yes | N/A | Yes | Yes | Yes | Yes | Yes | 7 | 100% |
| 11 | Balducci et al., 2017 (37) | Yes | No | N/A | Yes | Yes | Yes | Yes | Yes | 6 | 86% |
| 12 | Balkin et al., 2018 (38) | Yes | Yes | N/A | Yes | Yes | Yes | Yes | Yes | 7 | 100% |
| 13 | Bartczak & Oginska-Bulik, 2012 (39) | Yes | Yes | N/A | Yes | Yes | Yes | Yes | Yes | 7 | 100% |
| 14 | Bayhan Karapinar et al., 2019 (40) | Yes | Yes | N/A | Yes | Yes | Yes | Yes | Yes | 7 | 100% |
| 15 | Bonebright et al., 2000 (41) | Yes | Yes | N/A | Yes | Yes | No | Yes | Yes | 6 | 86% |
| 16 | Borges et al., 2021 (42) | Yes | Yes | N/A | Yes | Yes | Yes | Yes | Yes | 7 | 100% |
| 17 | Brady et al., 2008 (43) | Yes | Yes | N/A | Yes | Yes | Yes | Yes | Yes | 7 | 100% |
| 18 | Braun et al., 2019 (44) | Yes | Yes | N/A | Yes | Yes | Yes | Yes | Yes | 7 | 100% |
| 19 | Burke, 1999 (45) | Yes | No | N/A | Yes | No | No | Yes | Yes | 4 | 57.1% |
| 20 | Burke, 2000 (46) | Yes | No | N/A | Yes | No | No | Yes | No | 3 | 43% |
| 21 | Burke et al., 2003 (47) | Yes | No | N/A | Yes | No | No | Yes | No | 3 | 43% |
| 22 | Burke & Fiksenbaum, 2009 (48) | Yes | No | N/A | Yes | No | No | Yes | Yes | 4 | 57.1% |
| 23 | Caesens et al., 2014 (49) | Yes | No | N/A | Yes | Yes | Yes | Yes | Yes | 6 | 86% |
| 24 | Chamberlin & Zhang, 2009 (50) | Yes | Yes | N/A | Yes | Yes | Yes | Yes | Yes | 7 | 100% |
| 25 | Chang et al., 2022 (51) | Yes | Yes | N/A | Yes | Yes | Yes | Yes | Yes | 7 | 100% |
| 26 | Clark et al., 2014 (52) | Yes | Yes | N/A | Yes | Yes | Yes | Yes | Yes | 7 | 100% |
| 27 | Converso et al., 2019 (53) | Yes | Yes | N/A | Yes | Yes | Yes | Yes | Yes | 7 | 100% |
| 28 | Daniel et al., 2022 (54) | Yes | Yes | N/A | Yes | Yes | Yes | Yes | Yes | 7 | 100% |
| 29 | Del Libano et al., 2012 (55) | Yes | Yes | N/A | Yes | Yes | Yes | Yes | Yes | 7 | 100% |
| 30 | Di Stefano & Gaudiino, 2018 (56) | Yes | Yes | N/A | Yes | Yes | Yes | Yes | Yes | 7 | 100% |
| 31 | Dong et al., 2022 (57) | Yes | No | N/A | Yes | Yes | Yes | Yes | Yes | 7 | 100% |
| 32 | Eason et al., 2021 (58) | Yes | No | N/A | Yes | Yes | Yes | Yes | Yes | 6 | 86% |
| 33 | Falco et al., 2012 (59) | Yes | Yes | N/A | Yes | No | No | Yes | Yes | 5 | 71.4% |
| 34 | Falco et al., 2022 (60) | Yes | Yes | N/A | Yes | Yes | Yes | Yes | Yes | 7 | 100% |
| 35 | Flowers et al., 2000 (61) | Yes | Yes | N/A | Yes | No | No | Yes | Yes | 5 | 71.4% |
| 36 | Fujwara et al., 2016 (62) | Yes | Yes | N/A | Yes | Yes | Yes | Yes | Yes | 7 | 100% |
| 37 | Gillet et al., 2017 (63) | Yes | Yes | N/A | Yes | Yes | Yes | Yes | Yes | 7 | 100% |
| 38 | Gillet et al., 2018 (64) | Yes | Yes | N/A | Yes | Yes | Yes | Yes | Yes | 7 | 100% |
| 39 | Gillet et al., 2021 (65) | Yes | Yes | N/A | Yes | Yes | Yes | Yes | Yes | 7 | 100% |
| 40 | Gillet et al., 2022 (66) | Yes | Yes | N/A | Yes | Yes | Yes | Yes | Yes | 7 | 100% |
| 41 | Grawitch et al., 2017 (67) | Yes | No | N/A | Yes | Yes | Yes | Yes | Yes | 6 | 86% |
| 42 | Hakanen & Peeters, 2015 (68) | Yes | Yes | N/A | Yes | Yes | Yes | Yes | Yes | 7 | 100% |
| 43 | Hamilton Skurak et al., 2018 (69) | Yes | Yes | N/A | Yes | Yes | Yes | Yes | Yes | 7 | 100% |
| 44 | Hancock et al., 2019 (70) | Yes | Yes | N/A | Yes | Yes | No | Yes | Yes | 6 | 86% |
| 45 | Hauk & Chodkiewicz, 2013 (71) | Yes | Yes | N/A | Yes | Yes | Yes | Yes | Yes | 7 | 100% |
| 46 | Hirschi et al., 2019 (72) | Yes | Yes | N/A | Yes | Yes | Yes | Yes | Yes | 7 | 100% |
| 47 | Hogan et al., 2016 (73) | Yes | No | N/A | Yes | No | No | Yes | Yes | 4 | 57.1% |
| 48 | Huml et al., 2020 (74) | Yes | Yes | N/A | Yes | Yes | Yes | Yes | Yes | 7 | 100% |
| 49 | Huyghebaert-Zouaghi et al., 2021 (75) | Yes | Yes | N/A | Yes | Yes | Yes | Yes | Yes | 7 | 100% |
| 50 | Innanen et al., 2014 (76) | Yes | No | N/A | Yes | Yes | Yes | Yes | Yes | 6 | 86% |
| 51 | Kasemy et al., 2020 (77) | Yes | No | N/A | Yes | Yes | Yes | Yes | Yes | 6 | 86% |
| 52 | Kim et al., 2021 (78) | Yes | Yes | N/A | Yes | Yes | Yes | Yes | Yes | 7 | 100% |
| 53 | Kochanska et al., 2004 (79) | Yes | Yes | N/A | Yes | Yes | Yes | Yes | Yes | 7 | 100% |
| 54 | Kravina et al., 2010 (80) | Yes | No | N/A | Yes | No | No | Yes | Yes | 4 | 57.1% |
| 55 | Kravina et al., 2014 (81) | Yes | Yes | N/A | Yes | Yes | Yes | Yes | Yes | 7 | 100% |
| 56 | Lanaj et al., 2021 (82) | Yes | No | N/A | Yes | Yes | Yes | Yes | Yes | 6 | 86% |
| 57 | Levy, 2015a (83) | Yes | Yes | N/A | Yes | Yes | Yes | Yes | Yes | 7 | 100% |
| 58 | Levy, 2015b (84) | Yes | Yes | N/A | Yes | Yes | Yes | Yes | Yes | 7 | 100% |
| 59 | Loscalzo, 2021 (85) | Yes | Yes | N/A | Yes | No | No | Yes | Yes | 5 | 71.4% |
| 60 | Lundkvist et al., 2016 (86) | Yes | No | N/A | Yes | Yes | No | Yes | Yes | 5 | 71.4% |
| 61 | Mazzetti et al., 2019 (87) | Yes | Yes | N/A | Yes | Yes | Yes | Yes | Yes | 7 | 100% |
| 62 | McMillan et al.2004 (88) | Yes | Yes | N/A | Yes | Yes | Yes | Yes | Yes | 7 | 100% |
| 63 | McMillan & O’Driscoll, 2004 (89) | Yes | Yes | N/A | Yes | Yes | Yes | Yes | Yes | 7 | 100% |
| 64 | Molino et al., 2016 (90) | Yes | Yes | N/A | Yes | Yes | Yes | Yes | Yes | 7 | 100% |
| 65 | Molino et al., 2022 (91) | Yes | Yes | N/A | Yes | Yes | Yes | Yes | Yes | 7 | 100% |
| 66 | Morkevičiūtė & Endriulaitienė, 2022 (92) | Yes | Yes | N/A | Yes | Yes | Yes | Yes | Yes | 7 | 100% |
| 67 | Niehuis, 2007 (93) | Yes | Yes | N/A | Yes | No | No | Yes | Yes | 5 | 71.4% |
| 68 | Omar et al., 2018 (94) | Yes | Yes | N/A | Yes | No | No | Yes | Yes | 5 | 71.4% |
| 69 | Peplinska et al., 2015 (95) | Yes | No | N/A | Yes | Yes | Yes | Yes | Yes | 6 | 86% |
| 70 | Quinones-Garcia & Korak-Kakabadse, 2014 (96) | Yes | No | N/A | Yes | Yes | Yes | Yes | Yes | 6 | 86% |
| 71 | Rai et al., 2022 (97) | Yes | Yes | N/A | Yes | Yes | Yes | Yes | Yes | 7 | 100% |
| 72 | Reiner et al., 2019 (98) | Yes | Yes | N/A | Yes | Yes | No | Yes | Yes | 6 | 86% |
| 73 | Robinson & Carroll, 1999 (99) | Yes | No | N/A | No | No | No | Yes | Yes | 3 | 43% |
| 74 | Robinson, Carroll, & Flowers, 2001 (100) | Yes | Yes | N/A | Yes | No | No | Yes | Yes | 5 | 71.4% |
| 75 | Robinson, Flowers, & Carroll, 2001 (101) | Yes | Yes | N/A | Yes | Yes | Yes | Yes | Yes | 7 | 100% |
| 76 | Robinson & Kelley, 1998 (102) | Yes | Yes | N/A | Yes | No | No | Yes | Yes | 5 | 71.4% |
| 77 | Robinson & Kelley, 1999 (103) | Yes | No | N/A | Yes | No | No | Yes | Yes | 4 | 57.1% |
| 78 | Robinson & Post, 1995 (104) | Yes | No | N/A | Yes | No | No | Yes | Yes | 4 | 57.1% |
| 79 | Robinson & Post, 1997 (105) | Yes | No | N/A | Yes | No | No | Yes | Yes | 4 | 57.1% |
| 80 | Robinson et al., 2006 (106) | Yes | Yes | N/A | Yes | Yes | Yes | Yes | Yes | 7 | 100% |
| 81 | Ruiz-Garcia et al., 2022 (107) | Yes | Yes | N/A | Yes | Yes | Yes | Yes | Yes | 7 | 100% |
| 82 | Russo & Waters, 2006 (108) | Yes | Yes | N/A | Yes | Yes | Yes | Yes | Yes | 7 | 100% |
| 83 | Sawhney et al., 2022 (109) | Yes | Yes | N/A | Yes | Yes | Yes | Yes | Yes | 7 | 100% |
| 84 | Scafuri Kovalchuk et al., 2019 (110) | Yes | Yes | N/A | Yes | Yes | Yes | Yes | Yes | 7 | 100% |
| 85 | Schaufeli et al., 2008 (111) | Yes | No | N/A | Yes | Yes | Yes | Yes | Yes | 6 | 86% |
| 86 | Sharma & Sharma, 2018 (112) | Yes | No | N/A | Yes | Yes | Yes | Yes | Yes | 6 | 86% |
| 87 | Sheta & Hammouda, 2022 (113) | Yes | Yes | N/A | Yes | No | No | Yes | Yes | 5 | 71.4% |
| 88 | Shimazu & Schaufeli, 2009 (114) | Yes | Yes | N/A | Yes | Yes | Yes | Yes | Yes | 7 | 100% |
| 89 | Shimazu et al., 2011 (115) | Yes | Yes | N/A | Yes | Yes | Yes | Yes | Yes | 7 | 100% |
| 90 | Shimazu et al., 2014 (116) | Yes | Yes | N/A | Yes | Yes | Yes | Yes | Yes | 7 | 100% |
| 91 | Shimazu et al., 2020 (117) | Yes | Yes | N/A | Yes | Yes | Yes | Yes | Yes | 7 | 100% |
| 92 | Shin & Shin, 2020 (118) | Yes | No | N/A | Yes | No | No | Yes | Yes | 4 | 57.1% |
| 93 | Shkoler et al., 2017 (119) | Yes | Yes | N/A | Yes | Yes | Yes | Yes | Yes | 7 | 100% |
| 94 | Taheri et al., 2020 (120) | Yes | No | N/A | Yes | Yes | No | Yes | Yes | 5 | 71.4% |
| 95 | Tahir & Aziz, 2019 (121) | Yes | No | N/A | Yes | Yes | No | Yes | Yes | 5 | 71.4% |
| 96 | Taylor et al., 2019 (122) | Yes | No | N/A | Yes | Yes | Yes | Yes | Yes | 6 | 86% |
| 97 | Taylor et al., 2021 (123) | Yes | Yes | N/A | Yes | Yes | Yes | Yes | Yes | 7 | 100% |
| 98 | Therthani et al, 2022 (124) | Yes | Yes | N/A | Yes | No | No | Yes | Yes | 5 | 71.4% |
| 99 | Torp et al., 2018 (125) | Yes | No | N/A | Yes | Yes | Yes | Yes | Yes | 6 | 86% |
| 100 | Vedoato et al. 2020 (126) | Yes | No | N/A | Yes | Yes | Yes | Yes | Yes | 6 | 86% |
| 101 | Xie et al., 2022 (127) | Yes | Yes | N/A | Yes | No | No | Yes | Yes | 5 | 71.4% |
| 102 | Xu & Li, 2021 (128) | Yes | Yes | N/A | Yes | Yes | Yes | Yes | Yes | 7 | 100% |

*Note*. Possible answers: Yes, No, Unclear or N/A (Not Applicable). Checklist Items: 1. Were the criteria for inclusion in the sample clearly defined? 2. Were the study subjects and the setting described in detail? 3. Was the exposure measured in a valid and reliable way? 4. Were objective, standard criteria used for measurement of the condition? 5.Were confounding factors identified? 6. Were strategies to deal with confounding factors stated? 7. Were the outcomes measured in a valid and reliable way? 8. Was appropriate statistical analysis used? (129)
