# Supplementary material for: Work addiction and social functioning: A systematic review and five meta-analyses
Source: PLoS One. 2024 Jun 4;19(6):e0303563. doi: 10.1371/journal.pone.0303563 (PMC11149883; doi:10.1371/journal.pone.0303563)
Supplement: S3 Table — (DOCX) [file pone.0303563.s007.docx]

**S3 Table. Average effect sizes in the five meta-analysis and corresponding heterogeneity.**

|  | 1. Meta-analysis | 2. Meta-analysis | 3. Meta-analysis | 4. Meta-analysis | 5. Meta-analysis |
| --- | --- | --- | --- | --- | --- |
| **Average effect sizes** | **Moderate**  11 studies  ***r* = .338**  [0.23, 0.43]  *p* < .001 | **Small**  9 studies  ***r* = .274**  [0.16, 0.37]  *p* < .001 | **Small**  49 studies  ***r* = .284**  [0.21, 0.35]  *p* < .001 | **Small**  13 studies  **r = –.243**  [–0.356, –0.123]  *p* < .001 | **Small**  14 studies  **r = –.156**  [–0.218; –0.092]  *p* < .001 |
| **Heterogeneity** | *Q* (10) = 13.34  *p* < .001  *I^2^* = 91.18 | *Q* (26) = 48.66  *p* < .001  *I^2^* = 83.56 | *Q* (48) = 254.22  *p* < .001  *I^2^* = 97.87 | *Q* (12) = 137.60  *p* < .001  *I^2^* = 91.27 | *Q* (13) = 117.31  *p* < .001  *I^2^* = 88.91 |

*Note*. Significant effect sizes in bold.
