# Supplementary material for: Work addiction and social functioning: A systematic review and five meta-analyses
Source: PLoS One. 2024 Jun 4;19(6):e0303563. doi: 10.1371/journal.pone.0303563 (PMC11149883; doi:10.1371/journal.pone.0303563)
Supplement: S1 Fig — (DOCX) [file pone.0303563.s008.docx]

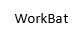


**S1 Fig. The number of specific work addiction measurement tools applied in the studies.**

*Note.* BWAS, Bergen Work Addiction Scale; CT, Compulsive tendencies subscale of the Work Addiction Risk Test; CWST, Children of Workaholic Parents Screening Test; DUWAS, Dutch Work Addiction Scale; DUWAS-10, 10-item version of Dutch Work Addiction Scale; MWS, Multidimensional Workaholism Scale; SNAP, Schedule for Adaptive and Nonadaptive Personality; SWBT, the Scale of Workaholism as Behavioral Tendencies; SZAP, Excessive Work Involvement Scale; WAQ, Workaholism Analysis Questionnaire; WART, Work Addiction Risk Test; WI-10, Work-Related Inventory; WorkBat, Workaholism Battery.
