# Supplementary material for: Work addiction and social functioning: A systematic review and five meta-analyses
Source: PLoS One. 2024 Jun 4;19(6):e0303563. doi: 10.1371/journal.pone.0303563 (PMC11149883; doi:10.1371/journal.pone.0303563)
Supplement: S2 Fig — (DOCX) [file pone.0303563.s009.docx]

**S2 Fig.** **Funnel plot of the first meta-analysis: The relationship between work addiction and work-life imbalance.**
