# Supplementary material for: Work addiction and social functioning: A systematic review and five meta-analyses
Source: PLoS One. 2024 Jun 4;19(6):e0303563. doi: 10.1371/journal.pone.0303563 (PMC11149883; doi:10.1371/journal.pone.0303563)
Supplement: S6 Fig — (DOCX) [file pone.0303563.s013.docx]

**S6 Fig.** **Funnel plot of the fifth meta-analysis: The relationship between work addiction and quality of** **relationships with friends, community, and colleagues.**
